# Development of a scalable single process for producing SARS-CoV-2 RBD monomer and dimer vaccine antigens

**DOI:** 10.3389/fbioe.2023.1287551

**Published:** 2023-11-17

**Authors:** Tammy Boggiano-Ayo, Julio Palacios-Oliva, Sumlai Lozada-Chang, Ernesto Relova-Hernandez, Jose Gomez-Perez, Gonzalo Oliva, Lourdes Hernandez, Alexi Bueno-Soler, Daidee Montes de Oca, Osvaldo Mora, Roberto Machado-Santisteban, Dayana Perez-Martinez, Beatriz Perez-Masson, Yanelys Cabrera Infante, Lisandra Calzadilla-Rosado, Yaima Ramirez, Judey Aymed-Garcia, Ingrid Ruiz-Ramirez, Yamile Romero, Tania Gomez, Luis A. Espinosa, Luis Javier Gonzalez, Annia Cabrales, Osmany Guirola, Kathya Rashida de la Luz, Franciscary Pi-Estopiñan, Belinda Sanchez-Ramirez, Dagmar Garcia-Rivera, Yuri Valdes-Balbin, Gertrudis Rojas, Kalet Leon-Monzon, Eduardo Ojito-Magaz, Eugenio Hardy

**Affiliations:** ^1^ Process Development Direction, Center of Molecular Immunology, Havana, Cuba; ^2^ Immunology and Immunobiology Direction, Center of Molecular Immunology, Havana, Cuba; ^3^ Quality Direction, Center of Molecular Immunology, Havana, Cuba; ^4^ Process Direction, Center of Molecular Immunology, Havana, Cuba; ^5^ Center for Genetic Engineering and Biotechnology, Playa, Cuba; ^6^ Finlay Vaccine Institute, Havana, Cuba

**Keywords:** SARS-CoV-2, RBD, CHO cells, perfusion culture, COVID-19, vaccine

## Abstract

We have developed a single process for producing two key COVID-19 vaccine antigens: SARS-CoV-2 receptor binding domain (RBD) monomer and dimer. These antigens are featured in various COVID-19 vaccine formats, including SOBERANA 01 and the licensed SOBERANA 02, and SOBERANA Plus. Our approach involves expressing RBD (319-541)-His6 in Chinese hamster ovary (CHO)-K1 cells, generating and characterizing oligoclones, and selecting the best RBD-producing clones. Critical parameters such as copper supplementation in the culture medium and cell viability influenced the yield of RBD dimer. The purification of RBD involved standard immobilized metal ion affinity chromatography (IMAC), ion exchange chromatography, and size exclusion chromatography. Our findings suggest that copper can improve IMAC performance. Efficient RBD production was achieved using small-scale bioreactor cell culture (2 L). The two RBD forms - monomeric and dimeric RBD - were also produced on a large scale (500 L). This study represents the first large-scale application of perfusion culture for the production of RBD antigens. We conducted a thorough analysis of the purified RBD antigens, which encompassed primary structure, protein integrity, N-glycosylation, size, purity, secondary and tertiary structures, isoform composition, hydrophobicity, and long-term stability. Additionally, we investigated RBD-ACE2 interactions, *in vitro* ACE2 recognition of RBD, and the immunogenicity of RBD antigens in mice. We have determined that both the monomeric and dimeric RBD antigens possess the necessary quality attributes for vaccine production. By enabling the customizable production of both RBD forms, this unified manufacturing process provides the required flexibility to adapt rapidly to the ever-changing demands of emerging SARS-CoV-2 variants and different COVID-19 vaccine platforms.

## 1 Introduction

The RBD (receptor-binding domain) is a critical subdomain of the SARS-CoV-2 (severe acute respiratory syndrome coronavirus 2) spike glycoprotein, which is responsible for COVID-19 (coronavirus disease 19) (Kirtipal et al., 2020). It contains 193 amino acids, four disulfide bonds (C336-C361, C379-C432, C391-C525, and C480-C488), and two N-glycosylation sites (N331 and N343) (Lan et al., 2020; Brun et al., 2021). RBD binds to the angiotensin-converting enzyme 2 (ACE2) receptor, enabling the virus to enter host cells such as respiratory and gastrointestinal epithelial cells (Shang et al., 2020; Wang et al., 2020).

The receptor-binding motif (amino acids 438-506) of RBD elicits neutralizing antibodies, making it a potent COVID-19 vaccine immunogen (Argentinian AntiCovid, 2020; Dai et al., 2020; Krammer, 2020; Kirtipal et al., 2020). RBD stimulates memory B cells, a broad T cell repertoire, and cross-neutralizing antibodies against SARS-CoV-2 and its variants, and is as effective as the full-length S protein in triggering cross-reactive neutralizing responses (Liang et al., 2022). RBD is also less likely to contain epitopes that could trigger autoimmune diseases (Dalvie et al., 2021; Pan et al., 2021; Sun et al., 2021).

RBD-based vaccines offer distinct advantages despite their suboptimal cytotoxic CD8^+^ T cell responses. They demonstrate enhanced safety by avoiding risks associated with genome integration and viral vector vaccines, as well as functioning efficaciously in immunocompromised individuals. Additionally, they are stable at temperatures between 4°C and 25°C, are readily producible with cost-effectiveness, and have been studied in detail (Sun et al., 2021; Eugenia-Toledo-Romani et al., 2022). RBD vaccines can quickly adapt in response to emerging mutations that increase ACE2 binding affinity (Kumari et al., 2022). Collectively, these attributes render RBD vaccines attractive for resource-constrained settings lacking infrastructure to support more complex vaccine platforms.

The small size and paucity of T cell epitopes, inadequate stability, and weak B cell receptor affinity of monovalent RBD implies suboptimal immunogenicity, necessitating multiple immunizations for effective immunity (Dai et al., 2020; Dong et al., 2020; Paco Pino et al., 2020; Pollet et al., 2021; Yang et al., 2021; Kumari et al., 2022; Liang et al., 2022). Therefore, several strategies have been developed to enhance the humoral response potency, breadth, and durability. These strategies include multimerization of RBD, conjugation or fusion with proteins, adjuvant utilization such as alum, and sustained antigen delivery via hydrogels (Pan et al., 2021; Valdes-Balbin et al., 2021; Yang et al., 2021; Heidary et al., 2022).

Using RBD as a SARS-CoV-2 vaccine antigen provides a safe, effective, and durable solution, with the added benefit of simplifying future vaccine development (Lee et al., 2021; Sinegubova et al., 2021). However, there are significant challenges related to RBD production and downstream processing, including adaptation to new RBD mutations, optimization of folding, disulfide bond formation, and post-translational modifications to ensure quality and efficacy (Chen et al., 2021; Krebs et al., 2021; Liang et al., 2022).

This report describes a single manufacturing process capable of producing both monomeric and dimeric forms of the RBD antigen suitable for varied COVID-19 vaccine formats. Chinese hamster ovary (CHO) cells expressed RBD resulting in over 30 mg/L of a mixture of RBD monomer and dimer in the fermentation supernatant. Cysteine at position 538 is ideal for conjugation with tetanus toxoid (for SOBERANA 02) or forming disulfide bridges (C538-C538) between two RBDs (for SOBERANA 01 and SOBERANA Plus) (Santana-Mederos et al., 2022). Both RBD monomer and dimer antigens can be purified with more than 96% purity and over 30% recovery, satisfying vaccine production quality standards based on different tests. These tests comprise mass spectrometry (MS), normal-phase high-performance liquid chromatography (NP-HPLC), size-exclusion chromatography (SEC)-HPLC, sodium dodecyl sulfate-polyacrylamide gel electrophoresis (SDS-PAGE), gel isoelectric focusing, circular dichroism, surface plasmon resonance (SPR), enzyme-linked immunosorbent assay (ELISA) (ACE-2 receptor binding assay), and mice immunization.

Having both RBD monomer and dimer antigens allows for flexibility in vaccine design. RBD monomer is used in some vaccines, whereas RBD dimer enhances the immune response in others. Utilizing a single process for producing both antigens provide economies of scale and adaptability to the SARS-CoV-2 variants.

## 2 Materials and methods

### 2.1 Genetic construct for expression of RBD (319-541)-His6 in CHO-K1 cells

The DNA sequence encoding the R319-F541 segment of the SARS-CoV-2 spike protein was assembled using a synthetic main fragment optimized for expression in CHO cells (*Crisetulus griseus*) and appropriate flanking oligonucleotides. The resulting gene was cloned into the pCMX-His intermediate vector via BssHII/NotI restriction sites, and the expression cassette was cloned into the pL6WBlast lentiviral vector via XhoI/EcoRV restriction sites. The resulting genetic construct, designated RBDsint201 (Supplementary Figure S1), was verified by sequencing. Additional information is provided in Supplementary Section 1.1.

### 2.2 Generation of stable cell lines and clones producing RBD (319-541)-His6

Human embryonic kidney 293T (HEK-293T) cells were transfected with the RBDsint201 genetic construct and auxiliary plasmids to produce lentiviral particles, which were used to transduce CHO-K1 cells. Oligoclonal cell lines producing high levels of biologically active RBD were selected and cloned by limiting dilution. A panel of ten clones from three and six rounds of lentiviral transduction were selected for small-scale (7 mL) expression studies. Experimental details are provided in Supplementary Section 1.2.

### 2.3 Production of RBD (319-541)-His6 in shake flask cultivation experiments

The best clones, three from each transduction schedule as described in Supplementary Section 1.2, were inoculated into 100 mL of serum-free media mixture MB02 and MB03 (50:50 ratio) (Merck, Germany) or HM04 (HiMedia, India) in 250 mL Erlenmeyer flasks (Corning Inc., USA) at a cell density of 0.5 × 10^6^ cells per flask. The cells were grown for 7 days in a Minitron orbital shaker incubator (Infors HT, Switzerland) operated at 120 rpm under conditions of 5% CO_2_ and 37°C. RBD was purified from the cell culture supernatant using Ni-NTA agarose beads (Qiagen, USA). Purified products were characterized by 10% SDS-PAGE and ELISA on ACE2-coated plates. All experiments were performed at least in duplicate to ensure reproducibility.

### 2.4 Effect of copper sulfate addition in shaking flask discontinuous culture

Clone 39-3x was cultured in 500 mL disposable Erlenmeyer flasks (Costar, USA) in a Minitron orbital shaker incubator (Infors HT, Switzerland) at 37°C in a 5% CO_2_ atmosphere in batch and semicontinuous mode. The culture medium used was a mix of MB02/MB03 (Merck, Germany), a protein-free medium, in a 1:1 ratio containing different molarities of copper (2.5, 5, 10, 35, 50 or 100 µM CuSO_4_). In the batch culture, cells were seeded at a density of 4 × 10^5^ cells/mL in 25 cm^2^ T-flasks. After 7 days of culture, the concentration of RBD dimer in the supernatant was determined using ELISA, utilizing a purified RBD dimer as a standard curve. On the other hand, flasks were seeded at 1 × 10^6^ cells/mL and allowed to grow for 24 h before starting the semicontinuous mode. The cell culture broth was centrifuged at 400 x g, 4°C for 10 min approximately every 24 h. The resulting supernatant was collected and stored at −20°C, while the cell pellet was suspended in 500 mL of fresh culture medium and returned to the Erlenmeyer flask, which was then placed on the incubation shaker. A sample was collected for cell counting, supernatant analysis, and RBD (319-541)-His6 purification each day before medium changes in the culture.

### 2.5 Production of RBD (319-541)-His6 in a 2 L-perfusion bioreactor

Long-term continuous perfusion cultures of CHO-K1 cells were carried out in a 2-liter bioreactor (Applikon Biotechnology, The Netherlands) equipped with a BioSep acoustic system (Applikon Biotechnology, The Netherlands) for cell retention. The bioreactor was inoculated with 0.3 × 10^6^ cells/mL or higher in a working volume of 1.8 L and operated in batch mode for 3–4 days. Perfusion was initiated once the cell concentration reached more than 2 × 10^6^ cells/mL. The cells were then fed with fresh media mixture MB02/MB03 (Merck, Germany). Temperature, pH and volumetric gas flow were carefully maintained within the range of 35–36.5°C, 6.5–7.2 and 0.0015–0.015 vvm of the total mixture (CO_2_, O_2_ and air), respectively. The stirring rate was set at 210–540 rpm (0.5–1.3 m/s), and the dilution rate was set at 0–1.5 medium volume per fermentation volume per day (vvd). Samples of culture broth (50 mL) were collected daily from the 2-liter bioreactor for analysis. Cell concentration and cell viability (i.e., how viable cells are distinguished from dead cells) were calculated using a Neubauer counting chamber and the trypan blue dye exclusion method.

### 2.6 Large-scale production of RBD (319-541)-His6 in a 750-liter perfusion bioreactor

Large-scale bioreactor cultures were set up in a 750-liter bioreactor (Bioengineering, Switzerland) with a twin rotofilter system for cell retention (Bioengineering, Switzerland). The bioreactor was inoculated with 0.5 × 10^6^ cells/mL in a volume of 500 L and kept under batch culture conditions for 48 h until the cell density exceeded 2 × 10^6^ cells/mL. Perfusion was then started at a dilution rate of 0.25 vvd up to 1.8 vvd. The increase in dilution rate is related to the cell specific perfusion rate (CSPR). To maintain the CSPR within a fixed range of 0.15–0.09 nL/cell/day, which was predetermined based on the culture medium and the performance of cells in a 2-liter laboratory bioreactor, discontinuous cell bleeding was performed to establish a steady state. Culture temperature (36.5°C ± 1°C), pH (6.95 ± 0.3), and maximum stirrer speed (70 rpm) were automatically monitored and maintained. Optical density was maintained at 50% ± 30% air saturation by a constant air flow of 0.004 L/min and addition of pure oxygen as needed up to 0.006 L/min. Bioreactor cultures were sampled daily for cell concentration, viability, and supernatant analysis. The harvest, which amounted to 1,000 L, was collected continuously using a Rotofilter separator.

The material balance equations describing the growth kinetics of cultures in perfusion included a leakage factor inherent to the device used for cell retention. The cell growth rate (µ) in h^-1^ was calculated using the following equation:

$$\frac{dVCD}{dt}=µ*VCD-Dc*VCD-\alpha *Dp*VCD$$

Where:

Dp: dilution rate in perfusion culture (h^-1^),

Dc: dilution rate in continuous culture (h^-1^),

α: leakage factor defined by the following equation

$$\alpha =\frac{VCD\left(DRC\right)}{VCD\left(B\right)}$$

Where:

VCD(DRC): Density of viable cells leaking through the cell retention device (cells/mL),

VCD(B): Viable cell density in the bioreactor (cells/mL).

Finally, the specific productivity (qRBD) in pg/cell/day (pcd) was determined using the equation:

$$\frac{d\left[RBD\right]}{dt}=qRBD*VCD-Dc*\left[RBD\right]-Dp*\left[RBD\right]$$

### 2.7 Small-scale purification of RBD (319-541)-His6

To purify the RBD protein from CHO-K1 cell-free culture supernatant, the supernatant was conditioned with 100 mM sodium phosphate buffer (pH 7.4) containing 1.5 M NaCl, 25 mM imidazole, and 50 µM copper sulfate, if copper sulfate was not already added to the culture medium. The RBD was first captured using a Chelating Sepharose Fast Flow immobilized metal affinity chromatography (IMAC) column (XL column) loaded with nickel ions (100 mM NiCl_2_). The IMAC chromatography was performed according to the manufacturer’s recommendations (see Supplementary Section 1.3). The RBD-containing IMAC eluate was then subjected to buffer exchange on a Sephadex G-25 column (Supplementary Section 1.3). Sephadex G-25-eluted RBD was subjected to cation exchange chromatography on an SP Sepharose Fast Flow column (Cytiva, USA) (Supplementary Section 1.3). To separate RBD monomer from RBD dimer, SEC-HPLC was performed using a Superdex 200 pregrade column (GE Healthcare, USA) (Supplementary Section 1.3). The purified RBD material was aseptically filtered through a 0.2 μm Sartopore 2 filter (Sartorius Stedim, Germany). It was then stored in a phosphate buffer solution containing Na_2_HPO_4_ (1.13 g/L) and KH_2_PO_4_ (0.35 g/L), along with NaCl (8.78 g/L) and trehalose (0.5 g/L), with a pH range of 7–7.4. The material was stored at −20°C until further analysis or use, such as in vaccine formulation. Recovery (mg/L) was calculated as the purified RBD mass divided by the harvested volume, while yield (%) was calculated as the purified RBD mass divided by the harvested RBD mass.

### 2.8 Large-scale purification of RBD (319-541)-His6

RBD in cell culture supernatant was purified using industrial chromatography columns. The purification process followed the same steps as previously described (Section 2.7), but on a larger scale, using a 30-liter IMAC (BPG) column, an 80-liter Sephadex G-25 column, a 2-liter SP Sepharose Fast Flow cation exchange chromatography column (Cytiva, USA), and a 45-liter Superdex 200 BPG column (GE Healthcare, USA) for SEC-HPLC.

### 2.9 Analytical techniques for characterization of the RBD (319-541)-His6

#### 2.9.1 SDS-PAGE

To evaluate the purity and oligomeric state of RBD (319-541)-His6, CHO-K1 cell supernatants (25 µL) or purified (5 μg) monomeric or dimeric RBD was separated on a 7% Tris/glycine SDS-PAGE gel under reducing (with dithiothreitol) and non-reducing conditions (Gallagher and Sasse, 1998). The gel was stained with Coomassie brilliant blue R250, destained with a mixture of methanol and acetic acid, and analyzed using a densitometer equipped with Image Lab software (Bio-Rad, USA) to determine RBD purity. In some experiments, electrophoresed gels were stained with silver as described elsewhere (Kavran and Leahy, 2014). In some experiments, RBD was also treated with PNGase-F (New England Biolabs, USA) under reducing conditions to remove N-glycans and loaded onto the gel to assess the effect of glycans on RBD size. Parallel gels were also transferred onto nitrocellulose membranes (Bio-Rad, USA) using a Mini Trans-Blot Electrophoretic Transfer Cell (Bio-Rad, USA) for Western blot analysis.

#### 2.9.2 Western blot for RBD (319-541)-His6

To confirm the presence and identity of RBD (319-541)-His6, nitrocellulose membranes were blocked with M-TBS/T (4% skim milk powder/0.1% Tween 20 in Tris-buffered saline [TBS]) overnight at 4°C. The blocked membranes were then incubated for 1 h at room temperature with polyclonal antibodies from convalescent COVID-19 patients (10 μg/ml M-TBS/T) to detect non-reduced RBD species. Other nitrocellulose membranes were incubated for 1 h at room temperature with an anti-His tag antibody (Sigma, USA) diluted 1:1,000 in M-TBS/T to detect reduced RBD. After incubation, the membranes were washed three times with TBS/T solution for 5 min each time. The appropriate anti-human/mouse immunoglobulin G (IgG) secondary antibodies conjugated to horseradish peroxidase (Sigma, USA) were then added at a final dilution of 1:1,000 in M-TBS/T and incubated at room temperature for 1 h. After the membranes were washed three times, the RBD bands were detected using a chemiluminescence kit (Santa Cruz Biotech, USA).

In some experiments, Western blot was used to analyze intracellular RBD. One million cells were harvested from 39-3x cell culture and centrifuged at 2000 x g for 10 min. The cell pellet was washed with PBS and then resuspended in 1 mL of ice-cold radioimmunoprecipitation assay buffer (cell lysis buffer, Merck-Millipore, USA) and incubated for 30 min. The lysed cells were centrifuged at 8000 *g* for 40 min at 4°C, and the supernatant was collected and stored at −20°C until the total protein was quantified by the bicinchoninic acid method (Hill and Straka, 1988). Supernatants (25 µL), purified RBD and lysed cell samples containing 30, 60, 90, and 120 µg total protein were separated by 10% SDS-PAGE followed by electroblotting (250 mA) onto a nitrocellulose membrane for 90 min. The membrane was blocked with low-fat milk in PBS-0.05% Tween 20 (PBST) before overnight incubation with ACE2-Fc (20 μg/mL). Immunodetection was performed with anti-human (γ-chain specific) horseradish peroxidase (1/2500 dilution) and Plus detection reagents (GE Healthcare, USA).

#### 2.9.3 Isoelectric focusing

IEF-PAGE analysis was performed using an automated horizontal electrophoresis device, “PhastSystem” (GE Pharmacia Biotech, USA), and dehydrated polyacrylamide gels (Phast Gel™ Dry IEF, Cytiva, Sweden). The gels were immersed in a mixture of Pharmalytes® IEF carrier ampholytes (86:14 [v/v]), pH 2.5–5:3–10) and 7 M urea. For all samples tested, 4 μg RBD (319-541)-His6 was applied per lane, and a pI range calibration kit (pH 3.5–9.3) was used to determine pI values (Amersham, GE Healthcare, UK). Operating conditions in the PhastSystem were 2.5 mA, 750 V at 15°C. After electrofocusing, the gels were stained with silver. Densitometric analysis of the gels was performed using a calibrated Syngene 900 imaging densitometer, and data processing was performed using GeneTools software version 4.3.8.0 (Syngene, Cambridge, UK).

#### 2.9.4 Human ACE2 binding ELISA

The ability of RBD (319-541)-His6 produced by CHO-K1 cells to bind human ACE2 was measured by ELISA. Ninety-six-well MaxiSorp Immuno ELISA plates (ThermoFisher, Waltham, MA, USA) were coated with 200 ng/well hACE-2-Fc (CIM, Cuba) in 0.1 M carbonate-bicarbonate buffer (pH 9.6) and incubated overnight at 4°C. After blocking with 200 μL/well of 2% nonfat dry milk in PBST at 37°C for 1 h, 50 μL of serially diluted samples (such as cell culture supernatants containing RBD or purified RBD species) in 0.2% nonfat dry milk/PBST buffer were added to the wells and incubated at 37°C for 1 h. 6× His-tagged PDL1 and purified recombinant RBD were used as negative and positive controls, respectively. An anti-SARS-CoV-2 RBD antibody (CIGB, Cuba) was added to the wells at 50 μL/well, followed by incubation at 37°C for 1 h. The plates were then washed 4 times with PBST. RBD bound to hACE-2-Fc on the plate was detected by adding 50 μL/well of 1:10000 diluted horseradish peroxidase-conjugated anti-mouse IgG monoclonal antibody (Sigma, USA) at 37°C for 1 h. After the ELISA plates were washed 5 times with PBST, the enzymatic reaction was visualized by adding 100 μL of 3,3′,5,5′-tetramethylbenzidine substrate solution (BD Biosciences, USA) to each well. The reaction was stopped by adding 100 μL of 1 M H_2_SO_4_ per well. After color development, absorbance was measured at 450 nm using a microwell system reader (Organon Teknika, The Netherlands).

#### 2.9.5 RP-HPLC

RP-HPLC was performed to separate RBD (319-541)-His6 samples using an Aeris 3.6 µm wide pore C4 column (Phenomenex, USA) heated to 37°C. Solvent A was 0.1% trifluoroacetic acid in water and solvent B was 0.1% trifluoroacetic acid in acetonitrile. A gradient from 0.5% to 100% B in 25 min was used to separate RBD samples. The flow rate was 1 mL/min. Detection of RBD was performed at 214 nm.

#### 2.9.6 Analytical SEC-HPLC

To determine the size and purity of purified RBD (319-541)-His6 molecular species, HPLC was performed using an instrument equipped with J-PU4086 pumps and a J-UV4070 detector (Jasco, Japan). Five μg of RBD was injected into a TSK 2000 or TSK 3000 column (7.8 mm × 30 cm) (Tosoh-biosciences, Germany) and eluted in 150 mM sodium phosphate (pH 7) at a flow rate of 0.25 mL/min. The elution of RBD species was monitored by detecting the absorbance at 280 nm.

#### 2.9.7 Normal phase-HPLC

The *N*-glycosylation profile of RBD (319-541)-His6 was determined as previously described (O’Flaherty et al., 2018). Briefly, 100 µg of RBD was treated with PNGase F (500 units, New England BioLabs, USA) at 37°C for 2 h to release N-glycans. The released N-glycans were then derivatized with 2-amino benzamide (2AB) by reductive amination at 65°C for 2 h. The resulting 2AB-labeled oligosaccharides were then separated by HPLC on a Prominence-Shimadzu instrument equipped with an Amide-80 column (TSKgel 250 × 4.6 mm, 5 μm, Tosoh BioScience, Japan) using a linear gradient from 20% of solution A (50 mM ammonium formate, pH 4.4) to 53% of solution B (pure acetronitrile). The 2AB derivatives were detected on-line by fluorescence using excitation (330 nm) and detection (420 nm) wavelengths. The retention time of each N-glycan peak was used to calculate GU values based on the use of HPLC-separated 2AB derivatives of a partially hydrolyzed dextran ladder as a reference. The GlycoStore database (https://glycostore.org/) was used for the structural assignment of the experimental GU values. Structures were represented based on the GlycoStore nomenclature for glycans.

#### 2.9.8 Electrospray ionization mass spectrometry

To analyze purified RBD (319-541)-His6 (monomer or dimer), 50 µg of protein in PBS was incubated with 5 mM N-ethylmaleimide for 30 min at room temperature (22°C). The RBD was then deglycosylated with 1 µL PNGase-F (New England Biolabs) for 2 h at 37°C to remove N-glycans. The eluate was desalted with ZipTips C18 (Millipore, USA) and analyzed by electrospray ionization-MS as described elsewhere (Espinosa et al., 2021). In addition, tryptic peptides generated by in-solution trypsin digestion of deglycosylated RBD were directly analyzed on a hybrid orthogonal QTof-2 tandem mass spectrometer (Micromass, Manchester, UK) for full sequence coverage and post-translational modifications as described previously (Espinosa et al., 2021). A signal was deemed informative for MS analysis if the signal-to-noise threshold was 2 or greater. Nonetheless, we were able to identify multiple C538 C-terminal peptides that underwent capping modification even if they produced lower-intensity signals below the signal-to-noise requirement of 2. This was achievable since we accumulated sufficient MS/MS spectra, resulting in improved combined MS/MS spectra for each relevant signal.

#### 2.9.9 Circular dichroism spectroscopy

The concentration of RBD (319-541)-His6 monomer and dimer samples was 0.1 mg/mL. Sample buffer was exchanged from PBS to MilliQ water using a NAP-5 column (Cytiva, USA). Far-UV circular dichroism (CD) spectra were obtained in the 190–250 nm range in 0.1 cm optical path quartz cuvettes using a J-1500 spectropolarimeter (Jasco, Japan). Recording was performed at 0.1 nm intervals, with a constant speed of 20 nm/min, 8 s response time, 1 nm bandwidth and 8 accumulations. The secondary structure content of the RBD was estimated by analyzing the deconvolution of the spectra using the BeStSel Internet server (http://bestsel.elte.hu/in-dex.php). Near UV-CD spectra were obtained in the 250–350 nm range in quartz cuvettes with a 1 cm optical path. The spectra were recorded at a constant speed of 100 nm/min, a response time of 1 s, 1 nm intervals, and 10 accumulations. Molecular fluorescence spectra were measured in the range of 300–450 nm, every 1 nm, with a response time of 1 s and 1 accumulation, using 0.1 cm optical path quartz cuvettes. Excitation was at 295 and 275 nm, using a 5 nm bandwidth for excitation and a 10 nm bandwidth for emission. RBD protein samples were at 0.15 mg/mL PBS.

#### 2.9.10 Surface plasmon resonance

SPR was performed using a BIACORE X sensor (GE Healthcare, USA) at 25°C in a multi-cycle mode to measure the affinity between the ACE2-mFc fusion protein and the recombinant RBD (319-541)-His6. Briefly, mFc-ACE2 was immobilized on a protein A biosensor chip (GE Healthcare, USA) through the flow cell 1 according to the manufacturer’s protocol. Flow cell 2 was used as a reference cell. The real-time response of RBD over the immobilized ACE2-mFc was recorded in duplicate in a concentration range from 1 to 2000 nM at a flow rate of 10 μL/min for 120 s, while dissociation took place for another 120 s. The running buffer was PBS (pH 7.2). After each cycle, the chip was regenerated with glycine buffer (pH 2). The equilibrium dissociation constant (binding affinity) was estimated with BIAevaluation® software (GE Healthcare, USA) using the Langmuir 1:1 interaction model. At least five curves were used for kinetic calculations.

#### 2.9.11 Immunogenicity in mice

Four groups of five BALB/c mice (age: 6–8 weeks, weight: 15–20 g) provided by the National Center for Laboratory Animal Breeding (CENPALAB, Havana, Cuba) were used. The immunogen, either monomeric or dimeric RBD (319-541)-His6, was administered by intramuscular injection on days 0 and 14. Sera were collected on days 0 (before immunization), 7 and 14. Mice in group 1 were inoculated with 10 µg monomeric RBD adjuvated with 500 μg Al(OH)_3_; group 2 with 10 µg dimeric RBD adjuvated with 500 μg Al(OH)_3_; group 3 with 10 µg monomeric RBD without adjuvant; and group 4 with 10 µg dimeric RBD without adjuvant. Titers of RBD-specific IgG were measured by ELISA. Plates (Nunc, USA) were coated with 50 μL of RBD monomer or dimer at 3 μg/mL in carbonate-bicarbonate buffer at pH 9.6 at 37°C for 1 h. Blocking was performed with 5% skim milk PBS at 37°C for 1 h. Plates were washed three times with PBS/Tween 20 and serum samples were added at serial dilutions from 1/100 to 1/1,000. Serum samples were diluted 1:3 v/v in PBS-1% bovine serum albumin solution, pH 7.2. After incubation at 37°C for 1 h and washing with PBS/Tween 20, goat anti-mouse IgG horseradish peroxidase antibody (Sigma-Aldrich) diluted 1/5000 in PBS-1% bovine serum albumin (pH 7.2) was added. After incubation at 37°C for 1 h and washing five times with PBS/Tween 20, 3′,3′5,5′-tetramethylbenzidine was added followed by incubation at 37°C for 20 min. The reaction was stopped with 2 N H_2_SO_4_ and the absorbance at 490 nm was measured using a microplate reader (ELISA Agilent Biotek, Thermo Scientific, USA). To report the results, the endpoint titer was defined as the highest reciprocal dilution of serum that yields an absorbance 2-fold higher than the absorbance of the pre-immune serum diluted at a 1/100 ratio. Logarithmic transformation was applied to ensure accurate analysis of the antibody titer data. To calculate the geometric mean titer, the logarithms of the five endpoint titers from the replicates were multiplied together. The fifth root of this product was then taken. The resulting value, representing the geometric mean titer, was then converted back to a real number by exponentiation for ease of interpretation and comparison. These calculations were carried out using the GEOMEAN function in Microsoft Excel (Microsoft, USA).

#### 2.9.12 Molecular dynamics simulation

To model the full-length structure of the mACE2-Fc, we used the RosettaCM application (Song et al., 2013) implemented in the Rosetta suite (Huang et al., 2011). The Fc region was modeled using the structure of the Fc fragment of the NIST IgG1 mAb described at 2.1 Å resolution (PDB id:5VGP) as a template (Gallagher et al., 2018). The ACE2 region was modeled using the structures of full-length human ACE2 in the presence of the neutral amino acid transporter B^0^AT1 with the RBD, at an overall resolution of 2.9 Å. The ACE2-B^0^AT1 complex is assembled as a dimer of heterodimers, with the collectrin-like domain of ACE2 mediating homodimerization (Yan et al., 2020) (PDB ID: 6M17). A short molecular dynamics simulation protocol was used to relax the linker region between the Fc domain and the ACE2 receptor. Simulations were performed using Gromacs 2022.3 (Abraham et al., 2015). Topologies of the system were built using the CHARMM-36 force field (Best et al., 2012). The mACE2-Fc protein was placed in a cubic box, with periodic boundary conditions, filled with TIP3P water molecules (Jorgensen et al., 1983). We checked that each atom of the proteins was at least at a distance of 1.1 nm from the box boundaries for all simulated systems. Each system was then minimized using the steepest descent algorithm. Next, relaxation of the water molecules and thermalization of the system were run in the NVT and NPT environments, each for 0.1 ns with a 2-fs time-step. The temperature was kept constant at 310 K with a v-rescale thermostat (Bussi et al., 2007). The final pressure was set at 1 bar using the Parrinello-Rahman barostat (Parrinello and Rahman, 1981). The LINCS algorithm (Hess et al., 1997) was used to constrain bonds involving hydrogen atoms. A cut-off of 12 Å was imposed to evaluate short-range non-bonded interactions. The Particle Mesh Ewald method was used to evaluate long-range electrostatic interactions (Darden et al., 1993). This method was used for all simulations performed.

#### 2.9.13 Statistics analysis

Where appropriate, data analysis was performed using Graphpad software (GraphPad Software, Inc, USA), and Student’s t-test and ANOVA were used as statistical tests. Significant differences between means were considered to exist when the *p*-value was less than 0.05.

## 3 Results and discussion

### 3.1 Expression of SARS-CoV-2 RBD (319–541) in CHO cells

SARS-CoV-2 RBD has been expressed in various eukaryotic cell hosts, including yeast, mammalian cells such as HEK-293 and CHO cells, genetically engineered insect cells, and thermophilic filamentous fungi (Argentinian AntiCovid, 2020; Chen et al., 2020; Dalvie et al., 2021; Klausberger et al., 2022; Sinegubova et al., 2021; Sun et al., 2021; Pan et al., 2021; Pollet et al., 2021; Yang et al., 2021; Nechooshtan et al., 2022). However, the presence of a free cysteine residue at position 538 in the RBD primary sequence poses a challenge for its expression due to disulfide bond scrambling (Klausberger et al., 2022). Several strategies have been developed to address this issue, including the removal of 538 (Chen et al., 2020; Sinegubova et al., 2021), a single mutation of C538 to an alanine residue (Pollet et al., 2021), or the construction of a fusion homodimer protein containing RBD (319-541) and IgG-Fc subunit (Pan et al., 2021). We developed a different strategy, which is explained below. All strategies, including ours, have demonstrated potential in producing high-quality RBD proteins for SARS-CoV-2 vaccine development.

### 3.2 Generation and characterization of RBD (319-541)-producing oligoclones

**FIGURE 1:**
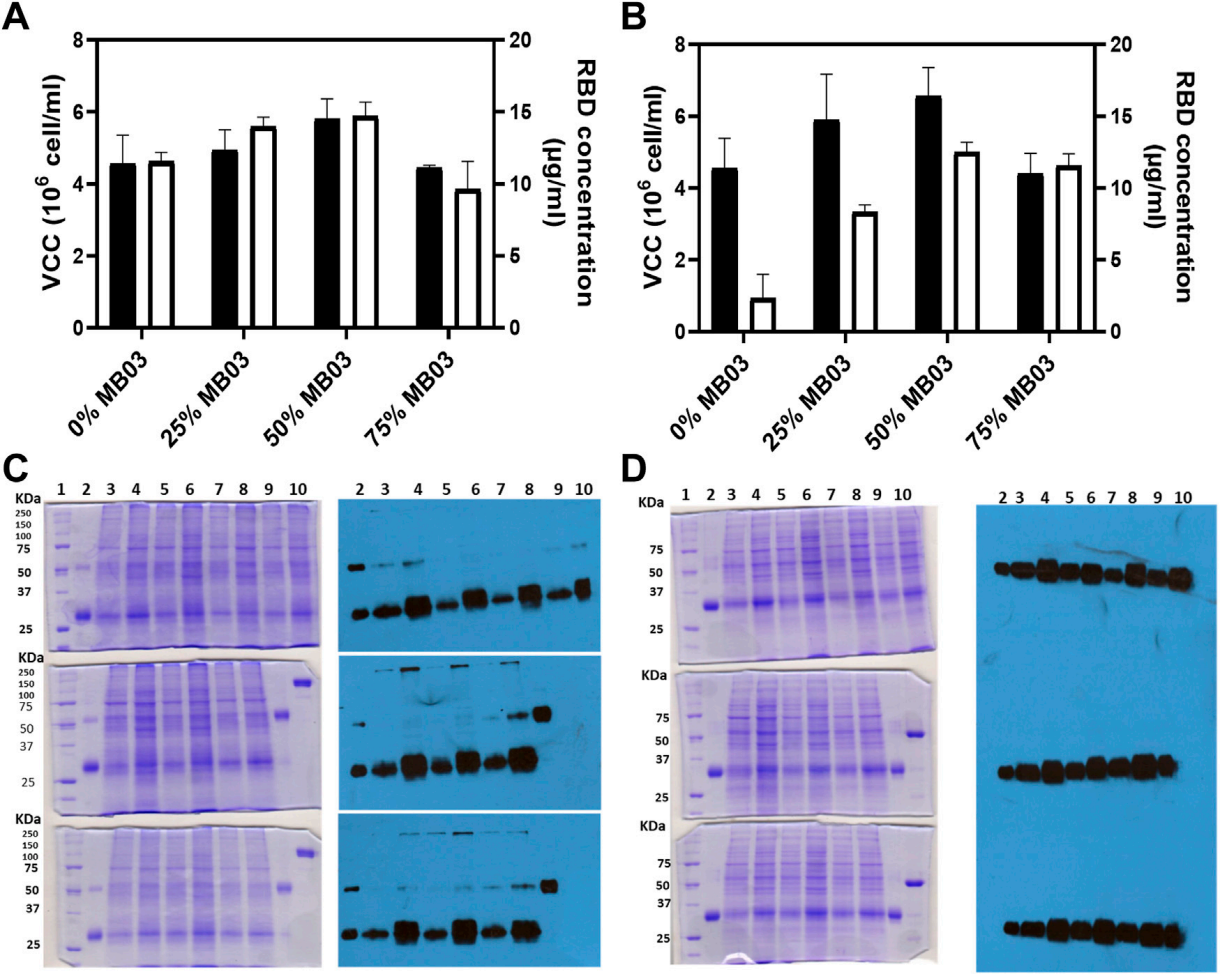
Panels **(A,B)** evaluate the effects of various culture media on RBD (319-541)-His6 concentration, measured by ELISA, and viable cell density (oligoclonal pool). Experiments were performed in T-flasks seeded at high density for 72 h. Panel **(A)** shows the combination of MB02 and MB03, while Panel **(B)** displays the mixture of HM04 and MB03. The black bars signify viable cell density, while white bars indicate RBD concentration. Panels **(C,D)** present the outcomes of 10% SDS-PAGE and Western blot of CHO-K1 culture supernatant (50 µL loaded), respectively. Panel **(C)** (first column) shows three Coomassie brilliant blue-stained SDS-PAGE gels electrophoresed under nonreducing conditions. The upper gel includes: lane 1, molecular mass marker; lane 2, RBD control, purified from the oligoclonal supernatant by IMAC; lane 3, oligoclone 4F2H; lane 4, oligoclone 4F2; lane 5, oligoclone 5F5H; lane 6, oligoclone 5F5; lane 7, oligoclone 2D11H; lane 8, oligoclone 2D11; lane 9, oligoclone 1B11H; and lane 10, oligoclone 1B11. The middle gel includes: lane 1, molecular mass marker; lane 2, RBD control as in the upper gel; lane 3, oligoclone 1E8H; lane 4, oligoclone 1E8; lane 5, oligoclone 3G2H; lane 6, oligoclone 3G2; lane 7, oligoclone 3D8H; lane 8, oligoclone 3D8; lane 9, RBD dimer (control), purified from oligoclonal supernatant by IMAC; and lane 10, RBD fused with the Fc domain of murine IgG1 (reduction control). The lower gel includes: lane 1, molecular mass marker; lane 2, RBD control as in the upper gel; lane 3, oligoclone 5B2H; lane 4, oligoclone 5B2; lane 5, oligoclone 4C9H; lane 6, oligoclone 4C9; lane 7, oligoclone 2D6H; lane 8, oligoclone 2D6; lane 9, RBD dimer (control) as in the middle gel; and lane 10, RBD-mFc (reduction control) as in the middle gel. The descriptor “H” in the clone codes denotes the use of MB03/HM04 culture medium; otherwise, the MB03/MB02 medium was used. Second column, three Western blots corresponding to the gels in C, visualized with an anti-RBD antibody. Panel **(D)** is similar to Panel **(C)**, except that the gels were electrophoresed under reducing conditions and the Western blots were visualized using an anti-His-tag antibody. The 4F2, 3D8, and 2D6 oligoclones expressed relatively high levels of RBD dimers.

To produce RBD in CHO-K1 cells, we employed a lentiviral transduction strategy that generated numerous oligoclones capable of producing detectable amounts of the recombinant RBD protein. Laboratory-scale production capacity was assessed by adapting oligoclonal cell lines to grow in suspension. Different proportions of culture media (MB02, HM04, and MB03) were evaluated to find the most suitable medium for cell growth and RBD expression. As depicted in Figures 1A, B, the density of oligoclonal cells increased with the addition of MB03 content (up to 50%), but declined when it was further increased to 75%. The highest concentration of RBD was attained when MB02 and MB03 were blended in equal amounts (Figure 1A). As the MB03 component increased by up to 50% in the combined medium (HM04 + MB03), the RBD concentration increased to above 12 mg RBD/L culture. The presence of MB03 resulted in a more than five-fold increase in RBD concentration compared to its absence (Figure 1B). Maximum cell densities were reached when the percentage of MB03 was 50% in both studies. Media MB02/MB03 (50:50, w/w) and HM04/MB03 (50:50, w/w) were selected for the next set of experiments to test oligoclones expressing RBD.

The RBD production capacity of CHO-K1 oligoclones was determined by growing them in the selected medium. Figures 1C,D show that the oligoclones were able to produce RBD in batch culture. Figure 1C, first and second columns, show that all oligoclones produced both monomeric (∼32,000 Mr) and dimeric (∼64,000 Mr) RBD (319-541)-His6, with more monomeric RBD being secreted. Total RBD expression (monomer + dimer) was consistent across all oligoclones, as shown in Figure 1D, second column. However, there were specific oligoclones (4F2, 3D8, 4C9, and 2D6) that demonstrated relatively higher levels of RBD expression, as depicted in Figure 1C (second column), in lanes 4 (upper image), 8 (middle image), 6 (bottom image), and 8 (bottom image), respectively. Figure 1D, first and second columns, revealed a single strong ∼32,000 Mr band, consistent with the anticipated size of the recombinant RBD protein, which included both the monomeric and reduced dimeric forms of RBD. Total RBD expression was higher in MB02/MB03 medium (Figures 1C, D, lanes 4, 6, and 8) than in HM02/MB03 medium (Figures 1C, D, lanes 3, 5, and 7).

**FIGURE 2:**
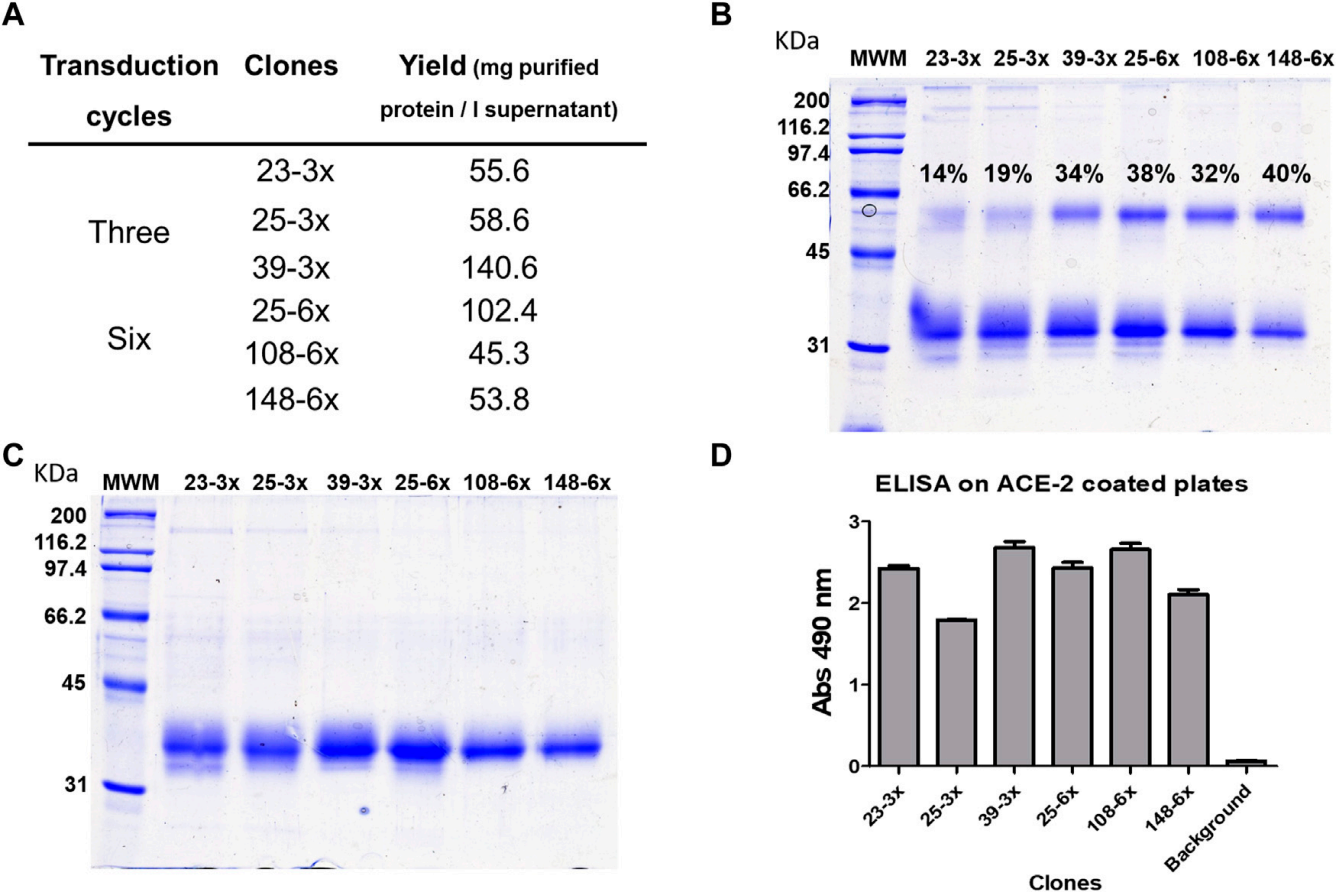
Comparison of RBD (319-541)-His6-producing clones obtained after lentiviral transduction of CHO-K1 cells. The six clones with the highest signals in the initial ELISA screening were adapted to grow in suspension in a mixture of 50% MB02 and 50% MB03. Three of the clones were derived from a three-round lentiviral transduction protocol, while the other clones were obtained after six rounds of transduction. Each clone was inoculated with the same number of cells (0.5 × 10^6^) into 100 mL of medium and incubated with agitation for 7 days. RBD was purified from the cell culture supernatant using Ni-NTA resin in a single chromatographic step. The total yields of purified RBD are shown in **(A)**. Analysis of the purified products by SDS-PAGE under non-reducing conditions revealed the presence of a mixture of RBD dimer and monomer. The percentages in the figure indicate the fraction of the total protein present in dimeric form. **(B)** Reduction of the disulfide bonds in the products resulted in a single band corresponding to the RBD monomer **(C)**. The biological activity and antigenicity of the RBD mixture (100 ng/mL) were demonstrated by ELISA on microtiter plates coated with the ACE2 receptor fused to human Fc **(D)**. The bound RBD was detected with anti-RBD S1 monoclonal antibody, which recognizes a conformational epitope, followed by anti-mouse IgG conjugated to horseradish peroxidase.

We characterized RBD-producing clones using suspension culture data from the oligoclone study and limiting dilution cloning. Figure 2 compares the three best clones obtained from three or six lentiviral transduction cycles and one limiting dilution cloning step. Clone 39-3x, obtained after three transduction cycles, proved to be the best RBD producer, yielding over 140 mg RBD/L of total IMAC-purified protein after 1 week of cell growth (Figure 2A). All clones generated a mixture of dimeric and monomeric forms of RBD, with clone 39-3x and clones acquired through six rounds of transduction producing over 30% dimeric RBD (Figure 2B). SDS-PAGE under reducing conditions revealed a single strong band with a molecular mass of 26,000 Mr, comprising both monomeric and reduced dimeric forms of RBD (Figure 2C). The IMAC-purified RBD from these clones was biologically active and antigenic based on ELISA results (Figure 2D).

To summarize, we generated RBD oligoclones and determined that MB02/MB03 medium was optimal for further experiments. Clone 39-3x was chosen for process development and scale-up over 6x-transduced clones to minimize the risk of genomic integrity loss and instability associated with repeated viral transduction events.

### 3.3 Identifying process development parameter that can improve the yield of RBD (319-541)-His6 dimer production

The insufficient yield of RBD dimers for developing vaccines based on them, such as SOBERANA 01 or SOBERANA Plus, prompted investigation into process development to increase their production by at least 50%.

#### 3.3.1 The RBD (319-541)-His6 dimer could be assembled extracellularly

Recombinant protein homodimers are typically assembled intracellularly in mammalian cells (Bustos-Valenzuela et al., 2010), but can also form in the cell supernatant through disulfide bonding, as shown with human E-cadherin’s EC1 domain expressed in *E. coli* (Trivedi et al., 2009). RBD homodimers have been obtained by tandem-repeat dimeric RBD (319-537) cloning (Yang et al., 2021) and an RBD (319-541)/Fc fusion protein approach (Pan et al., 2021; Sun et al., 2021). However, whether RBD (319-541)-His6 is intracellularly or extracellularly assembled is unclear and important to determine for increased RBD dimer production. To address this question, we conducted a Western blot analysis on the cell pellet and cell culture supernatant of clone 39-3x. Results in Supplementary Figure S2 suggest that most RBD homodimers form in the supernatant through the oxidation of the RBD sulfhydryl group at cysteine 538. This indicates that RBD homodimers are mostly formed extracellularly. However, future research should investigate whether potential disparities in the secretion efficiencies or protein stabilities between the two RBD assemblies might have an impact on these findings (Supplementary Figure S2).

#### 3.3.2 Cell culture viability influences the production of RBD (319-541)-His6

Cell lysis during CHO cell culture can affect heterologous protein quality, but it is unclear whether cell culture viability influences RBD-dimeric species generation (Kaneko et al., 2010).

**FIGURE 3:**
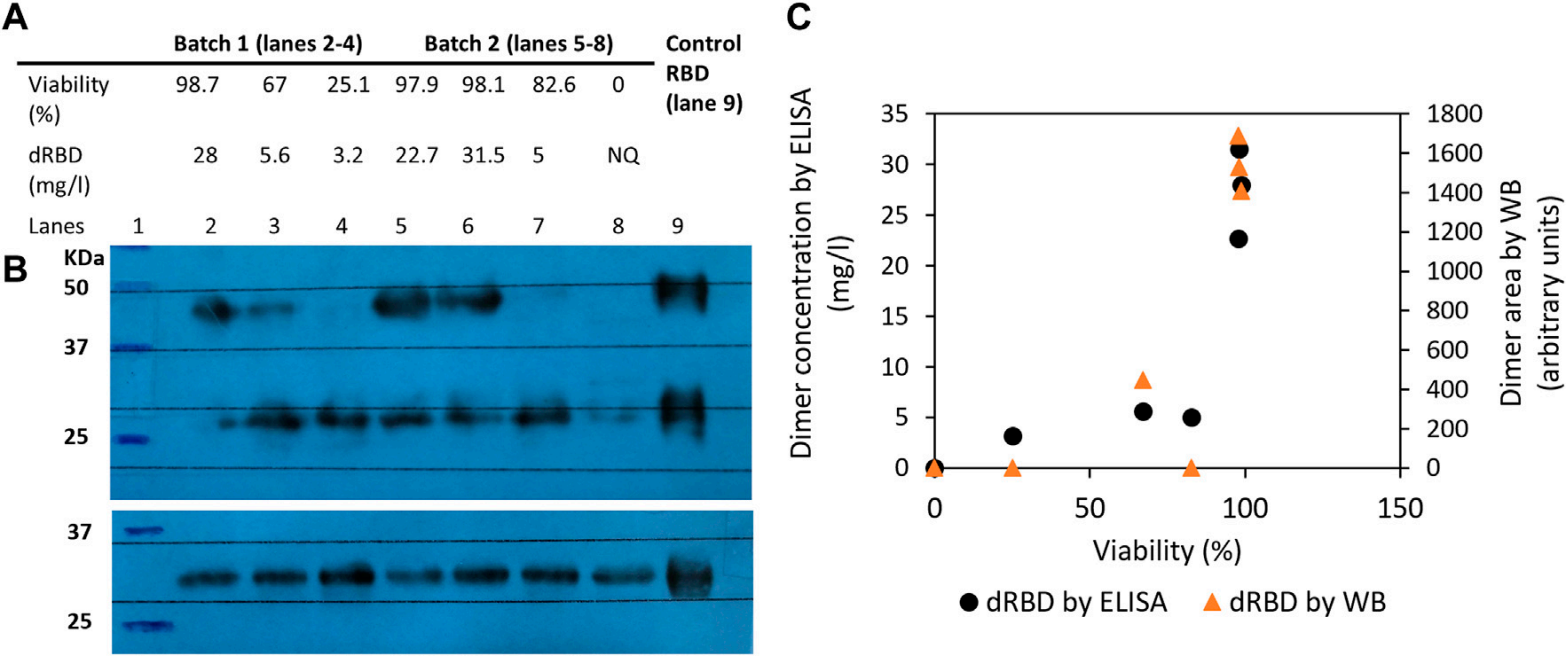
Influence of 39-3x cell culture viability on the resulting levels of RBD (319-541)-His6 dimers produced in the batch culture supernatant. In **(A)**, the concentration of dimeric RBD, as measured by ELISA and shown in the table, corresponds to the level of RBD dimers detected in the culture supernatant by non-reducing Western blot with an anti-RBD antibody (Panel **(B)**, top blot). The reduced Western blot (Panel **(B)**, bottom blot) was revealed with an anti-His tag antibody. In **(C)**, the area of dimeric RBD detected by both Western blot and ELISA is shown relative to cell viability.

We used ELISA and Western blot techniques to determine whether 39-3x cell line culture viability affects RBD dimer levels in the supernatant. Results showed that higher viability (above 97%) produced more dimeric RBD (above 20 mg/L) (Figure 3A, lanes 2, 5, and 6), while lower viability (below 83%) dramatically decreased dimeric RBD levels (below 6 mg/L) (Figure 3A, lanes 3, 4, and 7). Anti-RBD Western blot detected no dimers when the culture was no longer viable (Figure 3A, lane 8), although DTT-reduced samples had similar RBD amounts regardless of viability. Western blot densitometric analysis showed a decline in dimeric RBD as cell culture viability decreased (Figure 3C).

Besides reduced cell culture viability, RBD dimer instability at 37°C may have contributed to decreased dimeric RBD levels in batch culture. Dimeric RBD could decrease by over 20% after 7 days of storage at 37°C (data not shown). The batch cultures in this study lasted for 8–9 days, during which RBD in the supernatant was kept at 37°C, potentially degrading dimeric RBD. The combination of reduced viability and higher temperature may have negatively affected the proportion of dimeric RBD species.

Recapitulating, increased cell culture viability results in higher dimeric RBD levels, while decreased viability leads to lower levels.

#### 3.3.3 The RBD (319-541)-His6 dimerization may be enhanced by copper in the culture medium

Several strategies have been used to increase recombinant protein dimer production. Metz et al. (2017) immobilized sRecE, a recombinant flavivirus envelope protein, on a matrix for temperature-dependent generation of sRecE dimers. Transition metals, including copper, have been used to enhance cysteine oxidation in proteins like β-lactoglobulin (Bouhallab et al., 2004). Copper is an effective catalyst for cysteine oxidation, promoting disulfide bond formation in proteins through two reactions: (i) 2R-SH + O_2_^→^ R-SS -R + H_2_O_2_ and (ii) 2R-SH + H_2_O_2_^→^ R-SS-R + 2H_2_O. Copper supplementation (5–100 µM) in CHO cell cultures has been found to reduce the free thiol level of a humanized monoclonal antibody, thereby facilitating disulfide bond formation without causing significant toxicity to the cells (Chaderjian et al., 2005). Furthermore, copper supplementation (50 nM-50 µM) in culture medium increases CHO cell growth and IgG yield (Qian et al., 2011; Yuk et al., 2015).

**FIGURE 4:**
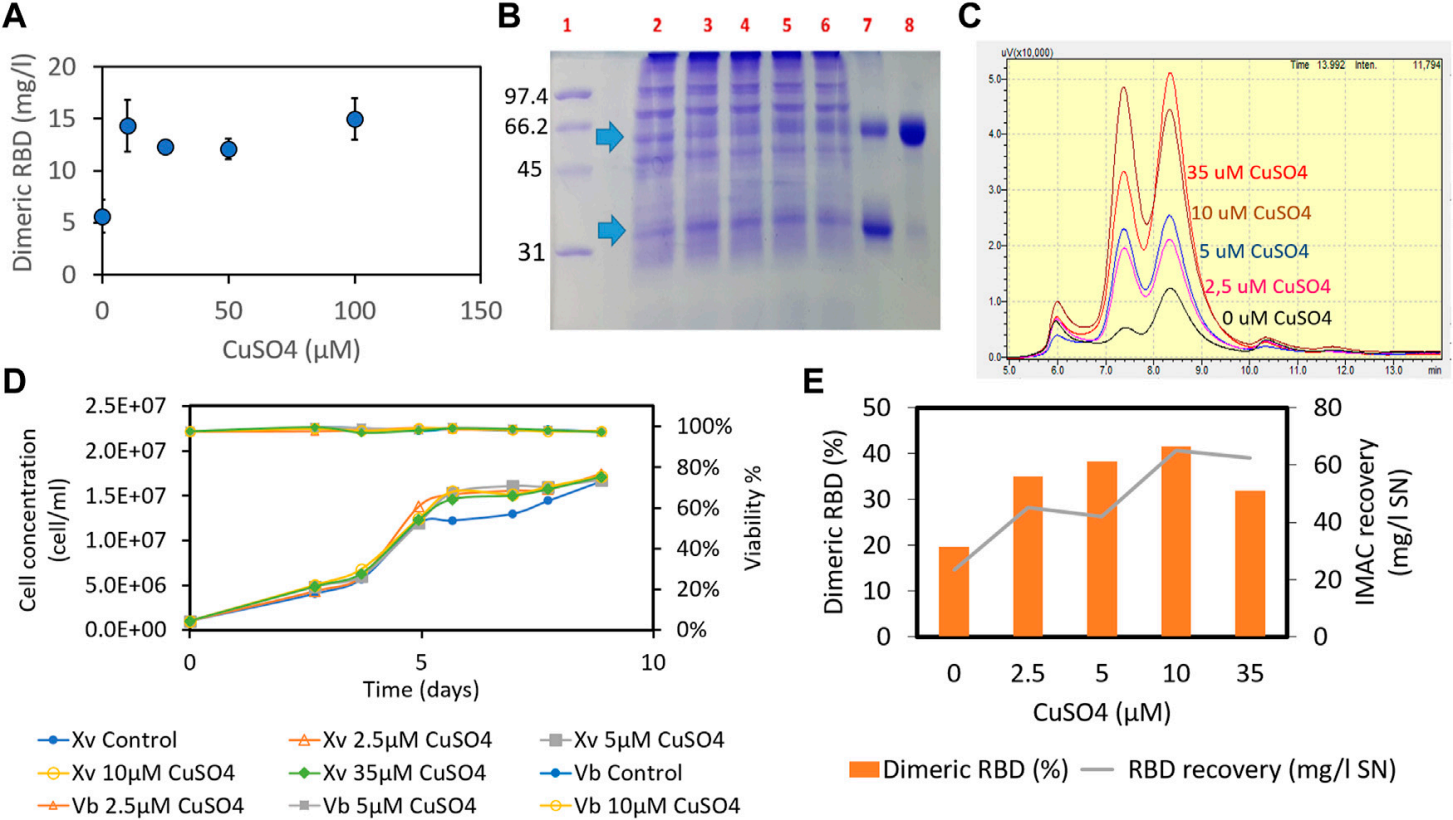
Effect of adding copper sulfate to the MB02/MB03 culture medium (1:1 ratio) on the formation of RBD (319-541)-His6 dimers. The effect of copper sulfate was tested in both batch and semicontinuous cultures. In the batch culture, CHO-K1 cells (clone 39-3x) were seeded at a density of 4 × 10^5^ cells/mL in 25 cm^2^ T-flasks. After 7 days of culture, the concentration of RBD dimer in the supernatant was determined using ELISA **(A)**. In the semicontinuous experiment, the 39-3x cells were cultured in 500 mL shake flasks at an initial seeding density of 1 × 10^6^ cells/mL. Starting on day 9 of culture, medium exchange was maintained at a dilution rate of 1vvd, and fresh medium was added daily. The culture supernatant was collected, pooled, and subjected to IMAC separation. In **(B)**, SDS-PAGE of the semicontinuous culture supernatant is shown, with arrows indicating the RBD monomer and dimer. Lane 1, molecular mass marker; lane 2, control culture of 39-3x; lanes 3-6, 39-3x cultures in the presence of different concentrations of CuSO_4_ (10 μM, 25 μM, 50 μM, and 100 µM); lane 7, a mixture of purified monomeric and dimeric RBD; lane 8, purified RBD dimer. **(C)** SEC-HPLC of IMAC eluates from the 39-3x clone semicontinuous culture harvest. **(D)** 39-3x cell concentration and viability profile of semicontinuous shake flask cultures with increasing concentrations of CuSO_4_ (2.5 µM, 5 μM, 10 μM, and 35 µM). **(E)** Recovery of total RBD and percentage of dimeric RBD from each IMAC separation for different amounts of CuSO_4_ added to the cell culture.

Having found that there is an insufficient proportion of the RBD dimer in the culture broth, we hypothesized that adding an oxidizing agent to the culture medium could further promote dimerization of the RBD (319-541)-His6 monomer under oxidative conditions. The addition of copper sulfate to the MB02/MB03 (50/50 ratio) culture medium at concentrations above 10 µM increased RBD dimer formation from 5.6 mg/mL to 14.3 mg/mL (Figure 4A).

Comparable results were seen in semicontinuous culture with cell retention across a range of copper concentrations from 2.5 to 35 μM, without adverse effects on cell growth or culture viability (Figures 4B–E). The cell culture reached a plateau after 5 days at a concentration of 15 × 10^6^ cells/mL and a dilution rate of 1vvd, while maintaining cell viability greater than 96% throughout the experiment (Figure 4D). Increasing copper sulfate concentration in the cell culture increased RBD dimers and total recovery from the IMAC gel (Figure 4E). Previous studies found a positive correlation between copper concentration in the culture medium and IgG title due to a twofold increase in the viable cell concentration resulting from metabolic shift, although there was also an increase in the specific production rate (Qian et al., 2011; Yuk et al., 2015). However, our experiments showed no difference in cell concentration between cultures with different levels of copper sulfate (Figure 4D), suggesting an increase in specific production rate. Adding 10 µM copper sulfate to the culture medium doubled the RBD dimer percentage to 41.5% and increased the total recovery of RBD from the IMAC gel to 65 mg/L (Figure 4E). We can speculate that copper-promoted oxidation of cysteine and reduced glutathione in the culture medium may counteract the C538 capping modification of RBD, thereby facilitating RBD dimerization (Zhong et al., 2017). In contrast to 10 µM CuSO_4_, adding 35 µM copper sulfate did not increase the recovery of the RBD dimer from the IMAC gel (Figure 4E). There seems to be a limit to the effect of copper on CHO culture performance, including productivity (Yuk et al., 2015). Yuk et al. (2015) found that above 13 nM copper sulfate, there was no further improvement in CHO culture performance. Optimal copper levels may vary depending on the cell line and culture process.

In short, copper sulfate enhances RBD (319-541)-His6 dimerization without affecting cell growth.

### 3.4 Establishing the purification process of RBD (319-541)-His6 monomer and dimer

Before implementing the three process parameters described in Chapters 3.3.1–3.3.3, we established a purification process for RBD (319-541)-His6. Various RBDs have been purified for research purposes using a combination of chromatography techniques (Paco Pino et al., 2020; Castro et al., 2021; Lee et al., 2021; Klausberger et al., 2022). IMAC chromatography with Chelating Sepharose Fast Flow is commonly used, but chelating species (e.g., EDTA and citric acid) in the harvest can interfere with the process (Supplementary Figure S3) (Lehr et al., 2000). Diafiltration can remove these species but is costly, time-consuming, and can also reduce the recovery of the target protein (Kittinger et al., 2020). However, Zhang et al. (2012) showed that adding cupric acetate (1.25–10 mM) to the harvest can improve the recovery of a heterologous enzyme from CHO cells without the need for diafiltration. In our experience, copper can improve the performance of IMAC chromatography for RBD (328-533)-His6, a model RBD protein that is only 17 amino acids shorter than RBD (319-541)-His6. Supporting evidence for this observation is provided in Supplementary Figure S4.

The purification process for RBD (319-541)-His6 is illustrated in Supplementary Figure S5A. It involves IMAC-to-SEC separation to remove protein oligomers and unrelated proteins, as shown in Supplementary Figures S5B–E. Three independent runs resulted in purified RBD monomer and dimer with a purity level exceeding 98.5%.

### 3.5 Perfusion cell culture facilitates the production of monomeric and dimeric species of RBD (319-541)-His6

RBD expression in mammalian cells is commonly performed in batch culture mode on a laboratory scale for molecular characterization or *in vitro* biological testing (Sinegubova et al., 2021; Arias-Arias et al., 2023). Transient systems have been the primary method for expressing RBD molecules (Castro et al., 2021; De March et al., 2022; Klausberger et al., 2022). For larger scale applications, such as immunogen for vaccine evaluation, RBD has been obtained from cultures grown in stirred tank bioreactors ranging from 10 to 250 L (Paco Pino et al., 2020; Pan et al., 2021). In these studies, cells were typically grown in fed-batch mode using protein-free medium. To our knowledge, there are no reports on the use of perfusion fermentation for RBD production.

Several factors were considered in the development of the fermentation process for RBD. Firstly, the potential advantages of using an on-site production platform that allows for high volumetric productivity in perfusion mode were considered, as discussed in Section 3.6. Secondly, the importance of maintaining high cell culture viability and minimizing RBD exposure to the culture to prevent potential degradation, as highlighted in Section 3.3.2, was recognized. Additionally, the potential benefits of supplementing the culture medium with copper to increase RBD dimer production, as discussed in Section 3.3.3, were taken into account.

**FIGURE 5:**
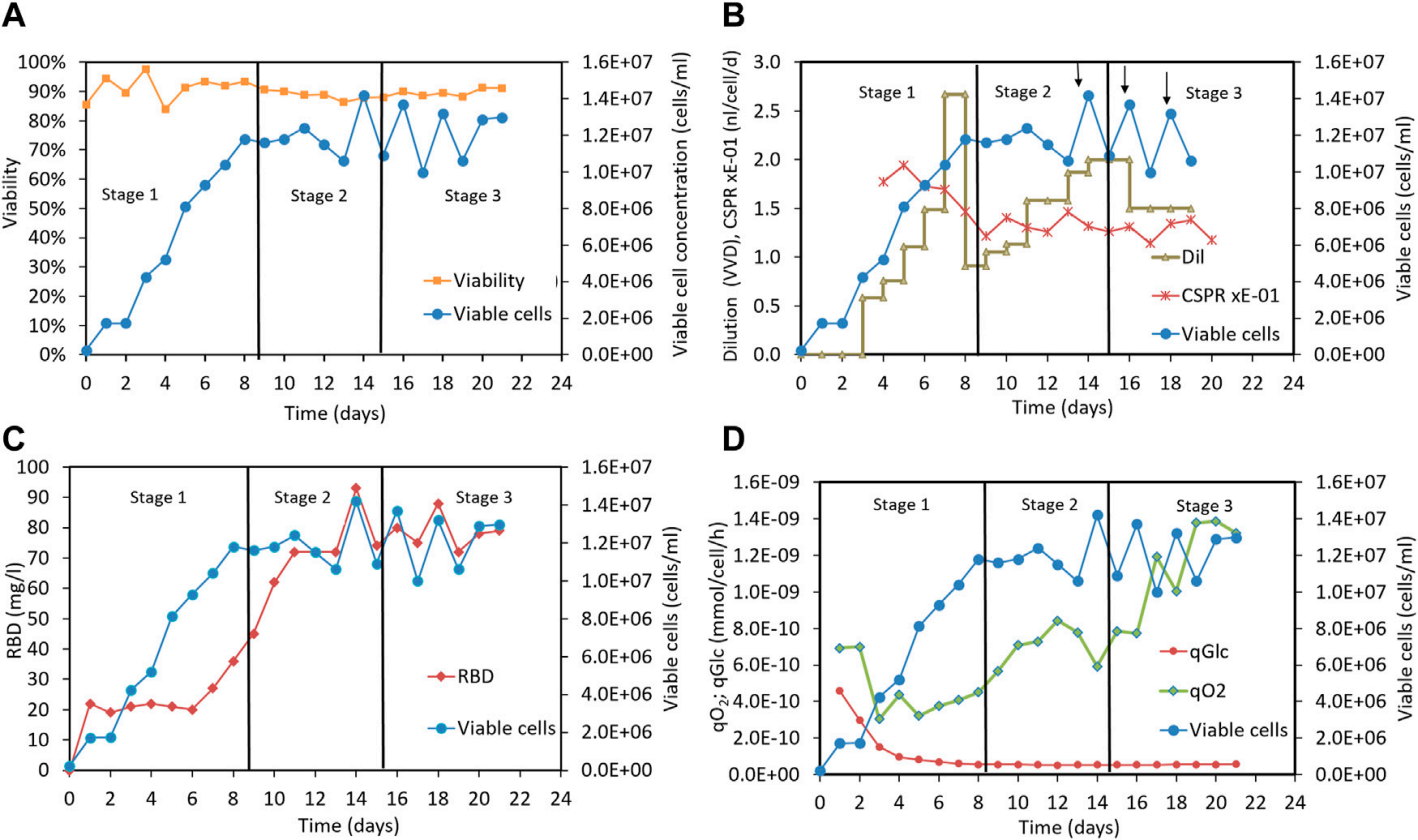
Perfusion culture of CHO-K1 cells for producing RBD (319-541)-His6 using a 2-liter bioreactor. **(A)** Time profiles indicating cell concentration and cell culture viability. **(B)** Time profiles indicating viable cell concentration, dilution rate, CSPR and cell bleeding (marked with arrows). **(C)** Time profiles indicating viable cell concentration and RBD concentration. **(D)** Time profiles indicating viable cell concentration, specific oxygen uptake rate and specific glucose uptake rate. Vertical lines are used to mark out the three different stages of the perfusion process: stage1 and stage 3, which do not involve Cu(II) addition, and stage 2, which includes Cu(II) addition in the culture medium.

To test these ideas, we established a 2-liter perfusion cell culture process using an acoustic cell separation device. The 39-3x cells were initially grown in batch mode until a viable cell concentration of approximately 2 × 10^6^ cells/mL was reached. The culture was then continuously fed with MB02/MB03 medium, and the supernatant containing the recombinant RBD was harvested through the cell separation device. The dilution rate in the perfusion culture was initially set to 0.5 vvd and then gradually increased to a maximum working value of 2.7 vvd. Figure 5 shows the typical profiles of various parameters during the perfusion cultures, and Supplementary Table S1 provides the mean values for each stage.

To facilitate the analysis, we divided the 22-day perfusion cycle into three stages, as shown in Figure 5. In the first stage, cells were cultured in MB02/MB03 medium without copper sulfate supplementation, and the dilution rate was increased to a maximum working value of 2.67 vvd. The viable cell concentration reached 12 × 10^6^ cells/mL and remained stable between 10 and 14 × 10^6^ cells/mL throughout the culture period, as depicted in Figure 5A. Cell viability remained above 85% after the cell culture reached stationary phase (Figure 5A). In the second stage, the cells were cultured in MB02/MB03 medium supplemented with 50 µM copper sulfate, and the dilution rate was maintained at 1-2 vvd to keep the CSPR stable around 0.13 nL/cell/d (Figure 5B). In the third stage, the cells were fed with MB02/MB03 medium without copper sulfate, and both the viable cell concentration and culture viability remained unchanged (Figure 5A).

As shown in Figures 5A, C positive correlation between RBD concentration and cell concentration was observed in stage 1, indicating that RBD production correlated with cell growth without copper supplementation. However, in stage 2, copper supplementation increased RBD concentration, eventually reaching up to 93 mg/mL, even when cell growth stabilized. This suggests that copper may enhance RBD production in a cell growth-independent manner, possibly by upregulating RBD-specific pathways or improving RBD secretion efficiency. These results highlight the potential of copper to decouple RBD production from cell growth and maximize titers in bioreactor perfusion cultures.

In stages 2 and 3, the volumetric productivity rate (Supplementary Table S1, column 11) and glucose uptake rate were similar (Supplementary Table S1, column 9), indicating comparable RBD production efficiency and glycolytic activity. However, the oxygen uptake rate was significantly higher in stage 3 (Supplementary Table S1, column 10), suggesting increased respiration and energy requirements to maintain the same productivity level. This could be due to increased cell stress, metabolic changes, or increased metabolism maintenance occurring when copper is removed from the culture medium. Interestingly, specific glucose consumption was unaffected by copper, suggesting that changes in central metabolism occurred downstream of glycolysis (Supplementary Table S1, column 9). The decoupling of oxygen uptake from RBD productivity in stage 3 suggests less efficient oxygen utilization for RBD production. These findings underscore the important role of copper sulfate in optimizing cell metabolism for efficient RBD production.

The clarified RBD-containing supernatants from each step of the perfusion process were purified, resulting in an overall yield of approximately 30% RBD for each purified supernatant. The proportion of RBD species (dimer and monomer) was measured in the elution of the cation exchange chromatography immediately before the final size exclusion chromatography step (Supplementary Figure S6). The copper-free materials from perfusion stages 1 and 3 exhibited very similar RBD dimer levels of approximately 23% (Supplementary Figure S6A) and 28% (Supplementary Figure S6C), respectively. In contrast, the copper-containing material from stage 2 showed an increase in RBD dimer levels up to 51% (Supplementary Figure S6B), which is consistent with previous laboratory-scale results using culture flasks, as discussed in Section 3.3.3. Small amounts of RBD oligomers remaining from the ionic chromatography step were separated and removed by the subsequent size exclusion chromatography step, as illustrated in Supplementary Figures S5D, E.

The viability of clone 39-3x remained above 85% at different agitation speeds (ranging from 0.5 to 1.3 m/s) and above 80% at different gas flow rates (ranging from 0.0015 to 0.015 vvm), indicating no significant impact on cell growth.

Overall, our study suggests that a perfusion cell culture process with copper supplementation can be a valuable tool for enhancing RBD dimer production and holds promise for larger scale RBD production.

### 3.6 RBD (319-541)-His6 monomeric and dimeric species can be produced on a large scale

We replicated the operating conditions of the small-scale perfusion process (Section 3.5) in a larger 500-liter bioreactor. This involved controlling various parameters, including impeller tip speed (1.4 m/s), maximum gas flow rate (0.01 vvm), dissolved oxygen content (50%), pH (6.5–7.1), temperature (35°C–36.5°C), medium composition, initial cell concentration (0.4 × 10^6^ cells/mL), and cell concentration at the beginning of medium feeding (2 × 10^6^ cells/mL). We adjusted the dilution rate to maintain viable cell concentrations and ensured a comparable D/Xv ratio (above 0.1 nL cel^−1^ d^−1^). To achieve the D/Xv ratio, the cell bleeding rate was set between 0.07 and 0.20 days^−1^ during the stationary phase. By maintaining similar operating conditions, our goal was to achieve scalable production of the RBD.

**FIGURE 6:**
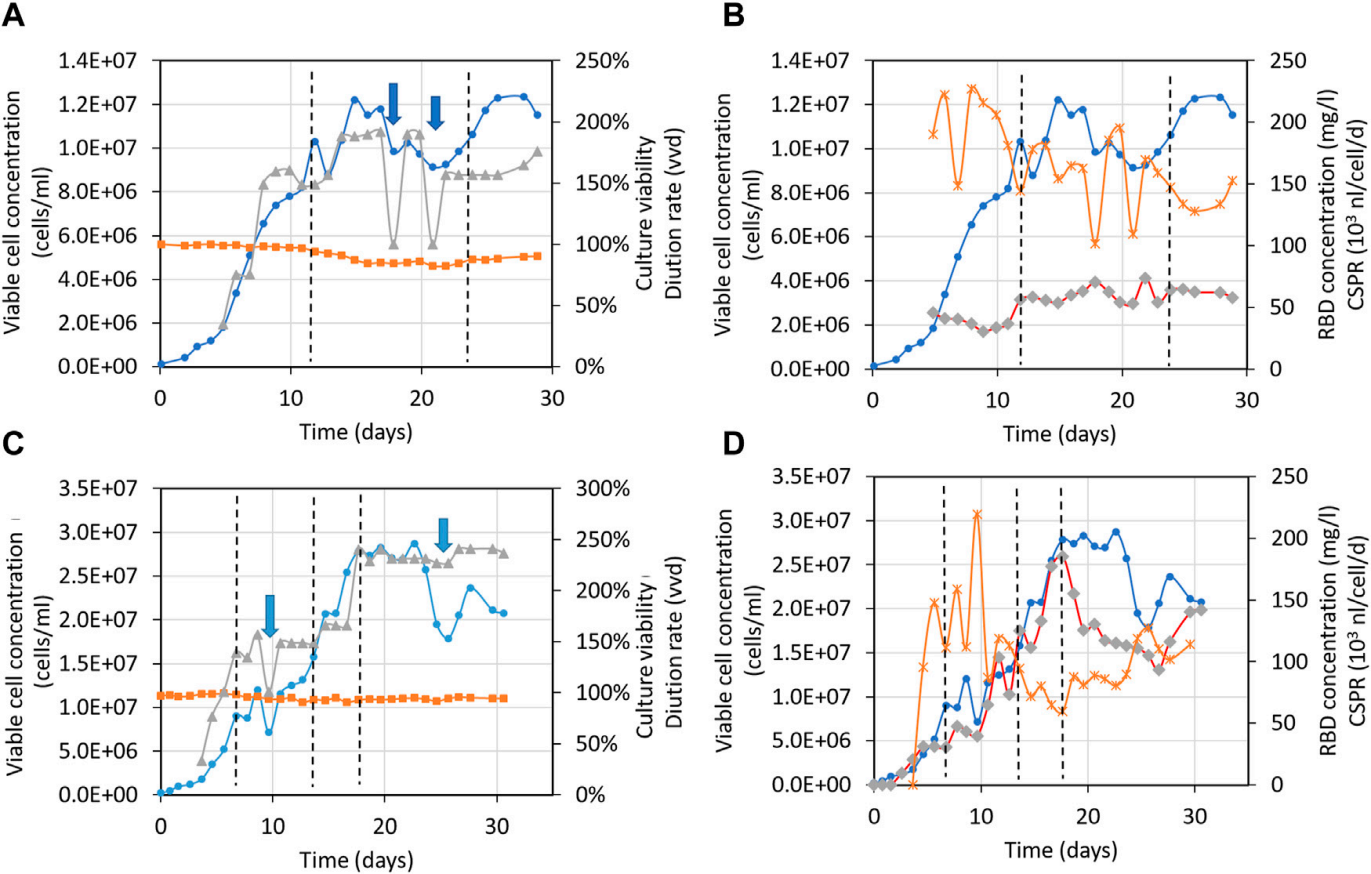
Time profiles of variables for the large-scale perfusion processes. Panels **(A,B)**, 500-liter bioreactor, run 1 (500LBR1). Panels **(C,D)**, 500-liter bioreactor, run 2 (500LBR2). Circles depict the 39-3x cell concentration, squares represent cell viability, triangles correspond to the medium dilution rate, stars indicate the cell-specific perfusion rate (CSPR), and diamonds represent the RBD (319-541)-His6 concentration. Dotted lines are used to demarcate the steady states during fermentation stages. Arrows indicate the cell bleeding procedure used to remove cells from the bioreactor.

Figures 6A–D displays time profiles of key variables from two independent perfusion processes carried out in a 500-liter bioreactor for the 39-3x clone. These profiles visually demonstrate the alterations in main parameters such as cell concentration, viability, and RBD concentration among others as time progresses. Table 1 (columns 3–5) presents the mean and standard deviation for these critical factors under steady-state conditions for the large-scale perfusion processes compared to a small-scale reference (Table 1, PP-run in column 2). This assessment enables us to evaluate the scalability of the production process.

**TABLE 1:** Parameters characterizing the large-scale perfusion process for RBD (319-541)-His6. Mean values and standard deviations for steady state are provided. The data were analyzed using one-way ANOVA and Tukey’s pairwise comparison at a 95% confidence level. Equal letters above the numbers indicate that there were no significant differences between groups.

Parameter	PP-run	500L BR-1	500L BR-2(ss1)	500L BR-2(ss2)
Dilution rate (d^−1^)	1.7 ± 0.1	1.6 ± 0.2	1.4 ± 0.2	2.34 ± 0.05
CSPR (nL/cel/d)	0.13 ± 0.09	0.16 ± 0.03	0.13 ± 0.04	0.09 ± 0.02
Cell bleed (d^−1^)	0.10 ± 0.06	0.07 ± 0.04	0.10 ± 0.08	0.20 ± 0.03
Viable cell concentration (cells/mL)	(12 ± 2) x 10^6 (a)^	(10 ± 2) x 10^6 (a)^	(11 ± 3) x 10^6 (a)^	(24 ± 4) x 10^6 (b)^
Viability (%)	90 ± 2^(a)^	88 ± 4^(a)^	94 ± 2^(b)^	94 ± 1^(b)^
RBD concentration (mg/L)	71 ± 13^(a)^	60 ± 6^(a)^	60 ± 16^(a)^	85 ± 16^(b)^
VP (mg/L/d)	129 ± 7^(a)^	100 ± 13^(b)^	85 ± 27^(b)^	212 ± 40^(c)^

Abbreviations: CSPR, cell specific perfusion rate; VP, volumetric productivity.

Table 1 (column 3) shows that the 500LBR1 process had similar performance to the 2-liter perfusion process under steady-state conditions, with comparable cell concentrations (10 and 12 × 10^6^ cells/mL, respectively), high cell viabilities (above 85%), and RBD concentrations (60 and 71 mg/L, respectively). The only minor difference was observed in volumetric productivity, with a value of ∼100 for the 500LBR1 process and 129 for the 2-liter process. In contrast, the 500LBR2 process (Table 1, columns 4 and 5) exhibited two distinct steady-states defined by the imposed medium dilution rate. The first steady-state, referred to as ss1 (D = 1.4 vvd), closely resembled the 500LBR1 process (D = 1.6 vvd), with an average cell concentration of 11 × 10^6^ cells/mL and an RBD concentration of 60 mg/L. To explore a new condition, we transitioned to a higher perfusion stage at D = 2.34 vvd (Table 1, column 5), resulting in even higher maximum cell densities (20-28 × 10^6^ cells/mL) and RBD concentrations. This change increased volumetric productivity from 85 mg/l/day (for ss1) to 212 mg/l/day (for ss2), demonstrating the process’s scalability and potential for further improvement.

Our process achieved a higher RBD concentration of 185 mg/L at the best operating conditions (Figure 6D) as compared to a previously reported concentration of 90 mg/L in a 5-liter stirred bioreactor (Castro et al., 2021). On average, we obtained 85 mg/L in the 500-liter bioreactor, demonstrating the high volumetric productivity of our process. These results make our production process suitable for clinical trials and practical application (Valdes-Balbin et al., 2021; Eugenia-Toledo-Romani et al., 2022; Santana-Mederos et al., 2022; Puga-Gomez et al., 2023).

The data presented in Figure 6 and Table 1 provide important insights into the performance of the RBD production process at different scales and conditions. These findings are valuable to refine RBD production and establish efficient strategies for large-scale manufacturing of COVID-19 vaccines.

### 3.7 Large-scale purification of RBD (319-541)-His6

**FIGURE 7:**
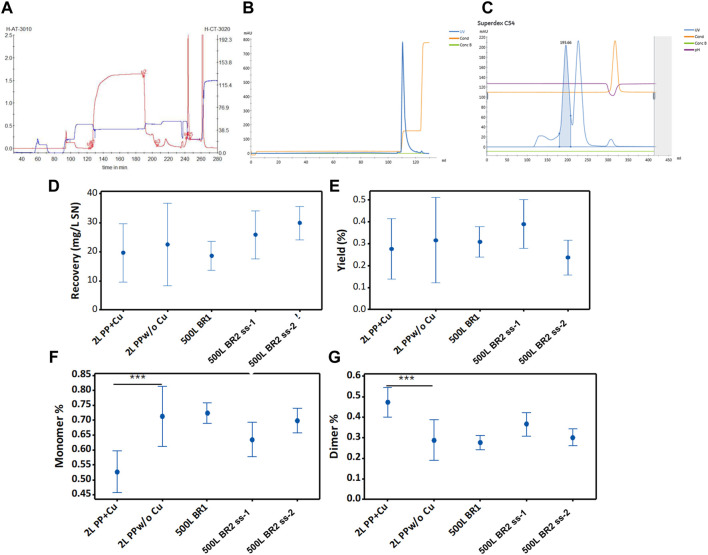
Purification of RBD (319-541)-His6 using different (independent) cell (39-3x) perfusion processes. **(A)** The initial purification step involves the capture of RBD present in the harvest of the 500-liter bioreactor. The chromatogram shows the separation of RBD by Chelating Sepharose FF with Ni(II) (25 L). The monitored signals comprise UV wavelength at 280 nm (in red) and conductivity (in blue). **(B)** Intermediate purification of IMAC-eluted RBD. The chromatogram corresponds to RBD separated on SP Sepharose FF (2 L). The signals monitored are UV wavelength at 280 nm (in blue) and conductivity (in orange). **(C)** Final purification of ion exchange purified RBD. The chromatogram illustrates RBD separated on Superdex 200 (45 L). The blue line represents the UV signal at 280 nm. The blue peak area corresponds to RBD dimer, and the uncolored next peak corresponds to RBD monomer. The monitored conductivity is shown in orange. Panels from **(D–G)** show data on total recoveries, yields, and percentages of RBD monomers and dimers, which are plotted for the 95% confidence interval of the mean parameter value.

Large-scale purification of the SARS-CoV-2 RBD has not been reported in the literature. However, the purification process described in Section 3.4 was successfully scaled up to purify RBD (319-541)-His6 from large-volume perfusions (Figures 7A–C). The recovery of RBD, defined as the total mass of RBD recovered from 1 L of culture supernatant, was approximately 19 mg RBD/L from the 500LBR1 perfusion process (Figure 7D). This recovery value is comparable to the recovery observed using the harvest from the 2-liter bioreactor (e.g., Figure 7D, 2L PP+Cu). With the second large-scale perfusion process (500LBR2, ss2), the recovery of RBD further increased to 30 mg RBD per liter (Figure 7D). This result is consistent with the higher average concentration of RBD in the 500LBR2 (ss2) process (85 mg/L) compared to the 500LBR1 process (60 mg/L).

The yield, defined as the ratio of eluted protein to the protein loaded onto the column, appeared to decrease in the 500LBR2 (ss2) process compared to the 500LBR1 process (Figure 7E). Nevertheless, statistical analysis (ANOVA, Turkey pairwise comparison) did not show a significant difference in yield between the two processes, suggesting a relatively small decrease. The high concentration of the 500LBR2 (ss2) in the IMAC column, increasing the RBD load, may have caused this slight decrease in yield. Factors such as resin saturation, protein competition for binding sites, and non-specific binding of impurities could contribute to a lower yield. The RBD yields obtained in this study (24%–40%) were lower than those reported for yeast-produced RBD (39%–50%) (Lee et al., 2021). It is important to note, however, that this comparison is limited because the RBD (332-549, C538A) produced by yeast has a reduced propensity for aggregation.

Without adding copper sulfate to the cell culture, approximately 60%–70% of the produced RBD protein existed in monomeric form (Figure 7F), while the remaining 30%–40% was present in dimeric form (Figure 7G). If necessary, the production of the RBD dimer can be increased by adding copper sulfate to the cell culture medium (Figure 7G, 2L PP+Cu).

Overall, the study showcases the successful large-scale purification of RBD, a crucial step in COVID-19 vaccine development. The recovery of RBD is influenced by culture conditions, emphasizing the importance of optimization. By manipulating these conditions, the production of different RBD forms can be tailored to meet specific downstream requirements.

### 3.8 Biochemical characterization of purified RBD (319-541)-His6

The purified RBD (319-541)-His6 preparations (Section 3.7) were thoroughly characterized using multiple assays to assess protein integrity, glycosylation, size, purity, structure, isoform composition, hydrophobicity, RBD-ACE2 interaction, functionality, and immunogenicity. These evaluations ensure the RBD’s quality and suitability for vaccine applications.

### 3.9 Aminoacid sequence of RBD (319-541)-His6 verified by ESI-MS/MS

Sequencing recombinant RBD proteins is a common practice to confirm their identity and detect any modifications or mutations that could affect their function. For instance, Arbeitman et al. sequenced RBD (319-537) expressed in *Komagataella phaffii (Pichia pastoris)* with 40% sequence coverage, and RBD (319-537) expressed in HEK-293 with 60% coverage (Argentinian AntiCovid, 2020). This helps ensure the RBD’s integrity and functionality for its intended use.

**FIGURE 8:**
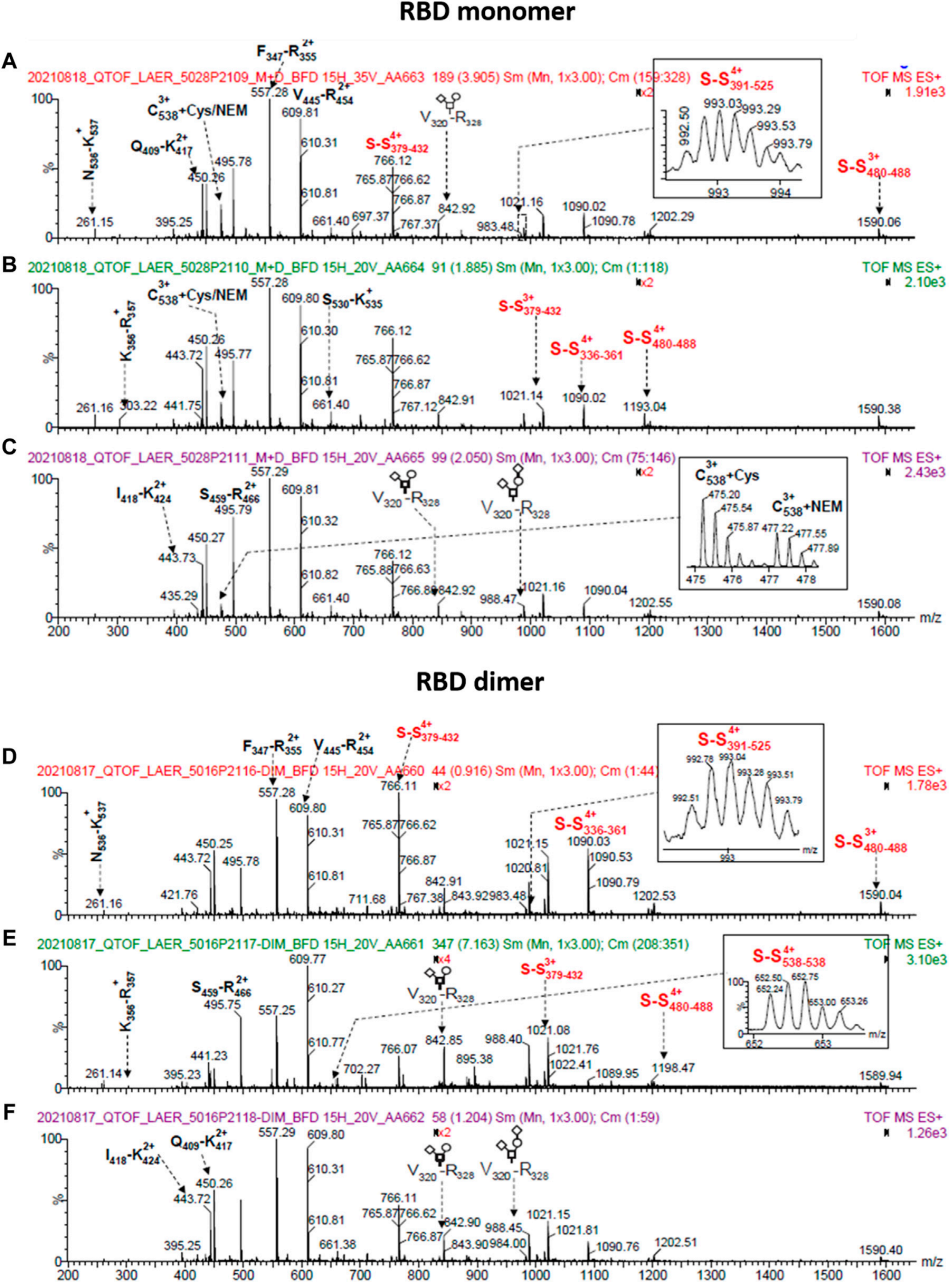
ESI-MS spectra of RBD (319-541)-His6 species produced on a large scale (500 L). The spectra correspond to samples that were deglycosylated with PNGase F in the presence of 5 mM N-ethylmaleimide and digested in solution with trypsin. The top panel displays spectra of RBD monomer from batches 5028/P2109 **(A)**, 5028/P2110 **(B)**, and 5028/P2111 **(C)**. The bottom panel shows spectra of RBD dimer from batches 5016/P2116 **(D)**, 5016/P2117 **(E)**, and 5016/P2118 **(F)**. The spectra insets provide magnified m/z regions, and the O-glycan structures conform to the SNFG (symbol nomenclature for glycans) system. The S-S notation refers to peptides with disulfide bonds.

To analyze the RBD (319-541)-His6 sequence, we utilized an in-solution buffer-free digestion method that has been shown to be effective with RBDs from various host cells (Espinosa et al., 2021). Supplementary Figures S1B, C show the expected amino acid sequences of the monomeric and dimeric RBD. Figure 8 displays the ESI-MS spectra of three large-scale batches of RBD monomers and dimers, respectively. Supplementary Table S2 provides the signal assignments for the ESI-MS spectra, covering 65% of the RBD amino acid sequence. The N-terminal (319RVQPTESIVR328) and the six C-terminal histidine residues (538CVNF541-HHHHHHH) of the RBD protein were identified (Supplementary Table S2, rows 1, 2, and 17). In the RBD monomer batches (5028/P2109, 5028/P2110 and 5028/P2111), both unmodified and modified C-termini with various structural groups were abundant at C538 (Supplementary Table S2, rows 17–41). In the RBD dimer batches (5016/P2116, 5016/P2117, and 5016/P2118), the C-terminus primarily appeared as a homodimer (C538-C538), as highlighted in red in Supplementary Table S3, row 6.

In summary, the amino acid sequence of RBD (319-541)-His6 was identified. In RBD dimers, the C-terminus forms a homodimer, whereas in RBD monomers, there is both a free and a modified C-terminus.

### 3.10 O-glycosylation at T323 and S325 of RBD (319-541)-His6 detected with ESI-MS

Out of the six potential O-glycosylation sites in SARS-CoV-2 RBD, only one site showed significant occupancy in RBD (305-543) expressed in HEK-293T cells (Bagdonaite et al., 2021). The occupancy of this site may be influenced by the solvent accessibility of T323, located close to the N-terminal region of RBD (305-543) in a flexible loop. Another study reported that T323 is O-glycosylated in over 90% of RBD (319-537) (Klausberger et al., 2022).

We confirmed the presence of O-glycosylation at the N-terminus of RBD (319-541)-His6 at T323 or S325 in all six analyzed batches, three of which were from RBD monomers and three from RBD dimers. As shown in Supplementary Table S4, different forms of the peptides 319RVQPTESIVR328 and 320VQPTESIVR328 were detected, including unmodified, HexNAc-modified, and HexNAc:Hex or HexNAc:Hex:NeuAc-modified forms. The most common form detected was glycosylation with sialic acid, including HexNAc:Hex:NeuAc, HexNAc:Hex+28Da:NeuAc, HexNAc:Hex:NeuAc2, and HexNAc:Hex+28Da:NeuAc2. The O-glycoform with HexNAc was only detected in one batch (5016/P2117), whereas the populations with HexNAc:Hex:NeuAc and HexNAc:Hex:NeuAc2 were found in all six batches with strong signals, suggesting they are the most abundant forms. Additionally, a HexNAc:Hex form with low abundance and a 190 Da residue, potentially a modified hexose, were identified.

Summing up, the RBD (319-541)-His6 protein undergoes glycosylation at T323 and S325 with a primary attachment of HexNAc:Hex:NeuAc and HexNAc:Hex:NeuAc2 O-glycoforms.

### 3.11 Intrachain and interchain disulfide bonds of RBD (319-541)-His6 detected with ESI-MS

The RBD (319-541)-His6 monomer contains nine cysteine residues, with eight of them forming four intramolecular disulfide bonds (Supplementary Figure S1B). In the RBD monomer, C538 is either free, modified (Supplementary Figure S1B and Section 3.12), or paired with another C538 residue in the RBD dimer form (Supplementary Figure S1C). These disulfide bonds help maintain the protein’s structure and stability. The presence of S-S bonds in the RBD protein remains consistent across various batches. The RBD monomer (batch 5028/P2109) and the RBD dimer (batch 5016/P2116) exhibit the expected four intramolecular S-S bonds, as demonstrated in Supplementary Table S3, columns 5 and 8. Likewise, Supplementary Table S3 columns 6, 7, 9, and 10 confirm the detection of S-S bonds in other protein batches. The C391-C525 disulfide bond was not observed in some batches (Supplementary Table S3, columns 6, 7, 9 and 10), possibly due to ionization suppression. The C538-C538 disulfide bond was observed in all three dimeric RBD batches (5016/P2116, 5016/P2117, and 5016/P2118 in Supplementary Table S3, row 6, columns 8-10). In contrast, the RBD monomer batches (5028/P2109 and 5028/P2110) did not exhibit detectable peaks at m/z 652.28 and 522.03, indicating the absence of the C538-C538 disulfide bond. However, we should mention that the signals indicative for C-terminal homodimerization were not exclusively observed in the dimeric RBD fraction. In the analysis, a peak at m/z 652.32 was detected in the batch 5028/P2111 (Supplementary Table S3, row 6, column 7), which could be attributed to RBD dimer impurity in the monomeric batch. Additionally, low-intensity signals (m/z 823.65, 4+ and m/z 659.13, 5+) were observed for a C-terminal disulfide bonded peptide (538CNVF541HHHHHH)-S-S-(387LNDLCFTNVYADSFVIR403), as indicated in Supplementary Table S3. The same disulfide bonded peptide was detected as low-intensity signals when the RBD monomer was digested with trypsin and analyzed by ESI-MS (Supplementary Table S2, m/z 823.65, 4+ and m/z 659.13, 5+). This suggests that the disulfide bonded peptide may have been a species that remained in the RBD monomer preparation due to incomplete purification.

In all analyzed batches, RBD species with an incorrect C391-C538 disulfide bond was found. This species likely represents an intrachain disulfide bond, as it was also detected in monomeric RBD samples (Supplementary Table S3, row 7).

In brief, the cysteine residues in the RBD protein form the anticipated disulfide bonds, as confirmed by ESI-MS analysis (Supplementary Figures S1B,C).

### 3.12 Chemical modifications of C538 are detected and identified by ESI-MS

C538 in the RBD protein was found to be S-alkylated with N-ethylmaleimide (Supplementary Table S2, row 17), while the other cysteines did not show any N-ethylmaleimide capping. The intense signal at m/z 477.21 (3+) confirmed the presence of RBD monomer with C538+ N-ethylmaleimide in the batches corresponding to the RBD monomer (5028/P2109, 5028/P2110, and 5028/P2111). However, this signal was weak in the batches corresponding to the RBD dimer (5016/P2116, 5016/P2117, and 5016/P2118), suggesting a lower population of RBD monomer in those batches.

Various chemical modifications were identified in C538, with a higher prevalence in the RBD monomer batches compared to the RBD dimer batches. This is typical of proteins with exposed free cysteines, where the capping of this amino acid with SH-containing species competes with interchain disulfide bond formation (Zhong et al., 2017). These chemical modifications, including cysteinylation, homocysteinylation, cyanation, cysteine dehydroalanine, glutathione addition, truncated glutathione addition, and other modifications of unknown structure, are indicated in red in Supplementary Table S2. This competition with interchain disulfide bond formation could decrease RBD dimerization as noted in this study (approximately 25%–30% dimerization). Similar findings have been reported for RBD (319-541) transiently expressed in HEK 293-6E cells (Klausberger et al., 2022).

We acknowledge that it was not possible to quantitatively estimate the extend of C538 disulfide scrambling or determine the relative abundance of the different C538 sulfhydryl modifications. This limitation arises from the use of the ESI-MS/MS technique based on the in-solution buffer-free digestion method employed in this study to characterize RBD variants, which is not currently quantitative for such analysis (Espinosa et al., 2021).

In short, S-alkylation of C538 in the RBD protein leads to multiple chemical modifications resulting from free cysteines being exposed. This ultimately leads to decreased dimerization.

### 3.13 N-glycosylation analyzed by NP-HPLC

The N-glycan molecules released from monomeric and dimeric RBD (319-541)-His6 by PNGase-F were analyzed using normal phase HPLC after derivatization with 2-aminobenzamide (Supplementary Figure S7). Both forms exhibited similar chromatograms, indicating shared glycosylated structures. Major peaks were observed at specific retention times (97.7 min, 100.1 min, 102.8 min, 109.6 min, and 120.8 min), including two main glycostructures (HexNAc2+Fuc+Hex3+HexNAc2 and one or two sialic acids). These structures accounted for approximately 56% of the total N-glycosylation. Longer retention time peaks may indicate the presence of more complex glycoforms with tri- and tetra-antennary structures, predominantly fucosylated, and varying in sialic acid content. Supplementary Table S5 provides the assigned glycan structures for peaks with longer retention times, based on a GU ladder calibration.

Different CHO cell lines producing RBD exhibit distinct N-glycosylation patterns, with our RBD having over 60% sialic acid, while another CHO cell line was reported to exhibit bi- and tri-antennary fucosylation with low levels of sialylation at N331 (2.5%) and N343 (0.8%) (Gstottner et al., 2021). These variations in N-glycosylation are likely attributed to differences in cell culture conditions, including the culture medium, fermentation mode, and CHO cell line used.

These findings provide insights into the glycosylation patterns of RBD (319-541)-His6, which can influence its function, stability and interactions with other molecules.

### 3.14 Size and purity of RBD (319-541)-His6 species determined by SEC-HPLC and SDS-PAGE followed by Western blot detection

**FIGURE 9:**
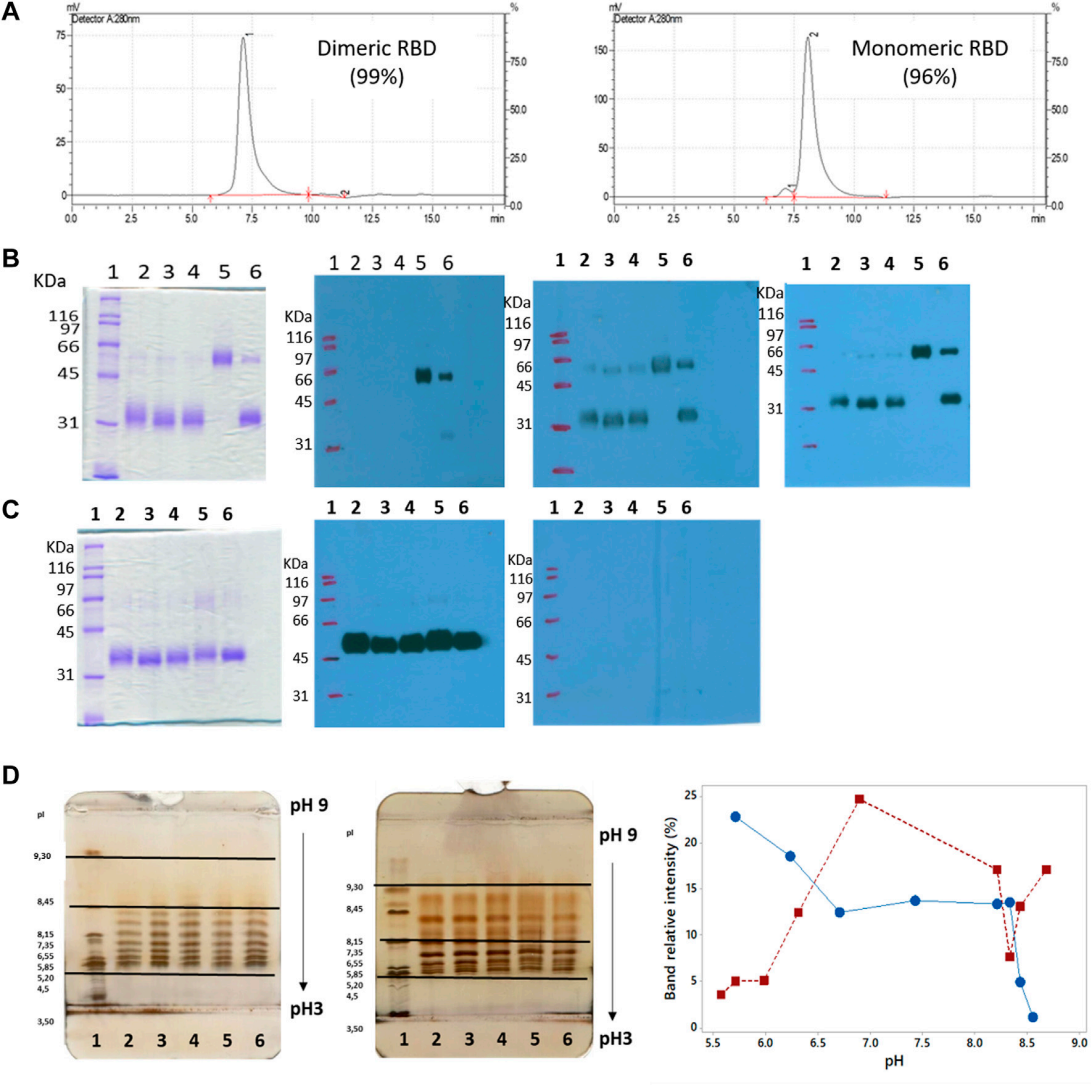
Size, purity, and isoforms of RBD (319-541)-His6. **(A)** SEC-HPLC chromatograms of purified dimeric and monomeric RBD. **(B)** Unreduced SDS-PAGE followed by Western blots probed with anti-His-tag antibody, anti-RBD antibody, and serum from convalescent COVID-19 patients. Lane 1, molecular mass marker; lanes 2-4, monomers of RBD; lane 5, RBD dimer; lane 6, monomeric and dimeric forms of RBD. **(C)** Similar to panel B, but the SDS-PAGE gel is run under reducing conditions. **(D)** Isoelectric focusing (IEF) of RBD. The left image shows the IEF of dimeric RBD. Lane 1, pH marker; lanes 2-6, RBD dimer. The center image exhibits the IEF of RBD monomer. Lane 1, pH marker; lanes 2-6, RBD monomer. The right image demonstrates the abundance of RBD isoforms as a function of pH focalization.

SEC-HPLC analysis demonstrated the high purity of both dimeric and monomeric forms of RBD. The dimer exhibited over 99% purity (Figure 9A, left), while the monomer showed a purity level over 96%, with less than 3% dimeric RBD present (Figure 9A, right), indicating the effectiveness of the purification process (Section 3.7) in isolating and enriching each RBD form.

Additional separation techniques were employed to support the findings. Non-reducing SDS-PAGE analysis of purified RBD samples revealed diffuse blue bands typical of glycosylated proteins, as seen in Figure 9B (lanes 2-6). Two bands were observed: a dominant band at approximately 32,200 Mr and a minor band at approximately 63,200 Mr, except for lane 5. The dominant band (32,200 Mr) was undetectable in Western blots (WB) when using an anti-His tag antibody, as shown in Figure 9B (first WB, lanes 2-4 and 6). In contrast, the minor band at 63,200 Mr was visible in Western blots when utilizing an anti-RBD antibody (Figure 9B, second WB, lanes 2-6) and polyclonal convalescent serum (Figure 9B, third WB, lanes 2-6). This confirmed that the minor band corresponded to the dimeric form of RBD. Treating RBD samples with dithiothreitol resulted in the appearance of a single band at approximately 36,000 Mr (Figure 9C, lanes 2-6), indicating the transition of the RBD dimer to a monomer under reducing conditions. These results support the high purity of the monomeric form of RBD, as observed in the SEC-HPLC analysis.

In essence, the purified RBD was highly pure, with both monomeric and dimeric bands observed in gel electrophoresis. The RBD dimer to monomer transition was confirmed by dithiothreitol treatment.

### 3.15 Secondary and tertiary structure of RBD (319–541)-His6 probed by circular dichroism

**FIGURE 10:**
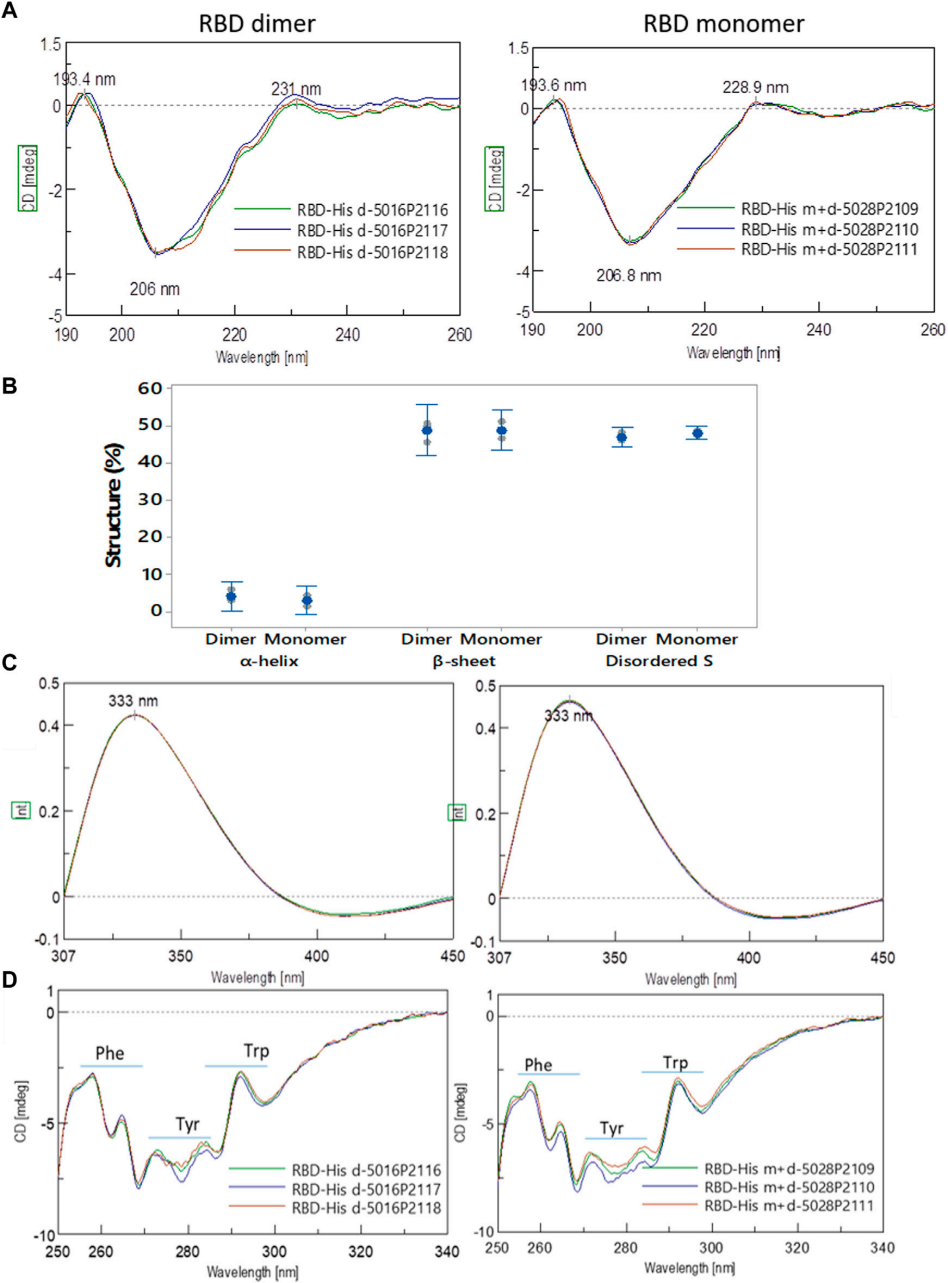
Spectroscopic characterization of RBD (319-541)-His6. **(A)** Secondary and tertiary structure of the RBD probed by far-UV circular dichroism (CD). The spectra of the RBD dimer are presented on the left, and the spectra of the RBD monomer are shown on the right. **(B)** The percentage of α-helix, β-sheet, and disordered random coil structures is indicated for both the RBD dimer and monomer. **(C)** Tryptophan fluorescence spectra are displayed for the RBD dimer on the left and the RBD monomer on the right. **(D)** The secondary and tertiary structure of the RBD is probed by near-UV CD.

Far-UV circular dichroism spectroscopy was used to analyze the purified RBD protein. The mean molar ellipticity per residue (deg.cm2.dmol^-1^) was measured as a function of wavelength (nm), which provided information about the secondary structure of the RBD protein, as detailed in Figure 10A. Both the monomeric and dimeric RBD showed similar spectra, with maxima at approximately 193.6 ± 0.7 nm and 230 ± 1 nm, and a minimum at around 206.5 ± 0.4 nm, indicating a similar secondary structure. The maximum and minimum emission values were consistent with previous reports for RBD (319-537) produced in HEK 293 and RBD (319-537) produced in *K. phaffii* (Argentinian AntiCovid, 2020), supporting the similarity in secondary structure. RBD spectra analysis using the BeStSel web server revealed that the protein consists mainly of β-sheet and random coil structures (45%–50%), with a smaller percentage of α-helices (4%–5%) (Figure 10B). The analysis is considered reliable based on the low normalized mean squared deviation value of 0.02–0.03.

The fluorescence spectra of dimeric and monomeric RBD in the 300–450 nm range, excited at 295 nm, exhibited overlapping peaks with a maximum at 333 nm (Figure 10 C), attributed to tryptophan residues. This emission maximum aligns with previous reports for RBD (319-537) (Argentinian AntiCovid, 2020), indicating consistency across studies. The intensity of tryptophan fluorescence was lower for RBD (319-545)-His6, suggesting less exposure to solvent. It could be that the tryptophan residues in RBD (319-545)-His6 are in a different environment compared to RBD (319-537). Nonetheless, the fluorescence spectra show comparable features, suggesting that the tryptophan residues hold a similar position within the protein structure.

Near-UV CD analysis of the tertiary structure of the RBD species (Figure 10D) revealed three distinct absorption regions: phenylalanine (255–270 nm), tyrosine (275–282 nm), and tryptophan (290–305 nm). Both the dimeric and monomeric RBD showed the same absorption maxima for phenylalanine (257.9 nm and 264.5 nm), tyrosine (272.9 nm and 284.2 nm), and tryptophan (291.9 nm), with no significant differences observed between the two forms of RBD (Figure 10D).

Summarizing, circular dichroism spectroscopy revealed similar secondary and tertiary structures in monomeric and dimeric RBD (319-541)-His6, providing valuable insights into RBD’s structural properties.

### 3.16 RBD (319-541)-His6 species separated by electrofocusing in gels

Although there is no reported isoelectric point for the RBD (319-541) dimer, studies have been conducted on the monomeric and trimeric charged variants using capillary isoelectric focusing (Krebs et al., 2021; Du et al., 2023).

We utilized gel isoelectrofocusing to analyze RBD (319-541)-His6. Monomeric RBD displayed nine bands within the pH range of 5.49 and 8.88 (Figure 9D, center gel image), while dimeric RBD showed eight bands within the pH range of 5.78 and 8.49 (Figure 9D, left gel image). Not only did the pH distribution range of RBD isoforms differ between the two RBD forms, but also their abundance. The proportion of acidic isoforms was greater in RBD dimer, while monomeric RBD primarily comprised neutral isoforms (Figure 9D, right gel image).

Differences in surface charge properties have been observed between monomeric and dimeric forms of molecules such as monoclonal antibodies (Plath et al., 2016). Monomeric RBD (319-541) from HEK-293 cells presents with a range of isoforms varying from 7.36 to 9.88 (Krebs et al., 2021). Our RBD (319-541)-His6 dimer has a narrower range of isoforms compared to the trimeric RBD (319-537) expressed in CHO cells (Du et al., 2023).

The isoform patterns of monomeric and dimeric RBD (319-541)-His6, observed in gel isoelectrofocusing, suggest differences in surface charge properties. These differences may affect RBD’s interaction with other molecules, including binding partners, as well as its overall biological activity. These differences could also impact the conformational stability, aggregation propensity, and antigenicity of RBD, potentially influencing vaccine efficacy. Further research is needed to fully understand the functional implications of these surface charge variations in RBD.

### 3.17 RBD (319-541)-His6 species separated by RP-HPLC

The RP-HPLC analysis in Supplementary Figure S8 shows that the dimeric form of RBD is more hydrophobic than the monomeric form. This aligns with previous findings of increased hydrophobicity in dimeric molecules like monoclonal antibodies compared to their monomeric counterparts (Plath et al., 2016).

The increased hydrophobicity of the dimeric RBD signifies a potential for greater aggregation or interaction with hydrophobic surfaces as compared to its monomeric counterpart. This could potentially impact its binding affinity to the ACE2 receptor or other molecules involved in viral entry, which is crucial for SARS-CoV-2 infection (Wang et al., 2020). Gaining an understanding of the surface properties of RBD (319-541)-His6 monomers and dimers is important in developing effective RBD-based vaccines. Further research is needed to explore the potential consequences of these differences on the biological activity of RBD, particularly in the context of SARS-CoV-2. Therefore, additional studies are required to investigate this hypothesis and unravel the structure-function relationship of RBD.

### 3.18 RBD (319-541)-His6-ACE2 interaction measured by surface plasmon resonance and ELISA

The affinity of RBD (319-541) for ACE-2 has been measured using surface plasmon resonance (Shang et al., 2020; Pan et al., 2021) and biolayer interferometry (Klausberger et al., 2022). The reported affinity constant values range from 1 to 49 nM.

We measured the response of immobilized ACE2 to increasing concentrations of the RBD (319-541)-His6 protein, including both monomeric and dimeric forms. The response signal, measured in resonance units, was found to be proportional to the concentrations of both the RBD monomer and dimer (Supplementary Figure S9). For the RBD monomer, the average association rate constant was (3.15 ± 0.57) × 10^5^ M^-1^⋅s^-1^, the dissociation rate was (8.14 ± 0.97) × 10^–3^ s^-1^, and the equilibrium dissociation constant was (26.6 ± 7.54) × 10^–9^ M. As for the RBD dimer, the average association rate constant was (6.60 ± 2.54) × 10^5^ M^-1^⋅s^-1^, the dissociation rate constant was (11.8 ± 3.06) × 10^–3^ s^-1^, and the equilibrium dissociation constant was (18.3 ± 2.3) × 10^–9^ M. Both the RBD monomer and dimer bound ACE2 with nanomolar affinity, typical for this interaction (Sui et al., 2014; Lan et al., 2020; Wang et al., 2020). Regardless of the RBD conformation, the SPR data were well fit by the 1:1 Langmuir interaction model with drift baseline, suggesting that a single RBD binds one ACE2 molecule, as shown in Table 2. The good fit (chi squared ≤1.66) confirms the appropriateness of this binding model (Table 2).

**TABLE 2:** Kinetic parameters estimated by fitting experimental surface plasmon resonance data (Supplementary Figure S9) to the Langmuir 1:1 interaction model (chi-squared ≤1.66). The interaction between the monomeric and dimeric RBD (319-541)-His6 and ACE2 receptor is characterized by the association rate, dissociation rate, maximum resonance units, and affinity constant. The data were obtained from three independent batches of RBD monomer and dimer.

Sample	Ka (M^−1^ ⋅ s^−1^)	Kd (s^−1^)	Rmax (RU)	K_D_ (M)	Chi^2^
_m_RBD (25/10/21)	3.14E+05	7.23E-03	90.7	2.30E-08	1.66
_m_RBD (28/10/21)	2.59E+05	9.16E-03	74	3.53E-08	1.24
_m_RBD (01/11/21)	3.72E+05	8.04E-03	97.7	2.16E-08	1.44
_d_RBD (25/10/21)	5.24E+05	10.8E-03	12.7	2.06E-08	0.47
_d_RBD (28/10/21)	9.53E+05	15.2E-03	12.1	1.59E-08	0.86
_d_RBD (01/11/21)	5.02E+05	9.31E-03	9.99	1.85E-08	1.25

Abbreviations: Ka, association rate; Kd, dissociation rate; Rmax, maximum resonance units; K_D,_ affinity constant; m, monomer; d, dimer.

**FIGURE 11:**
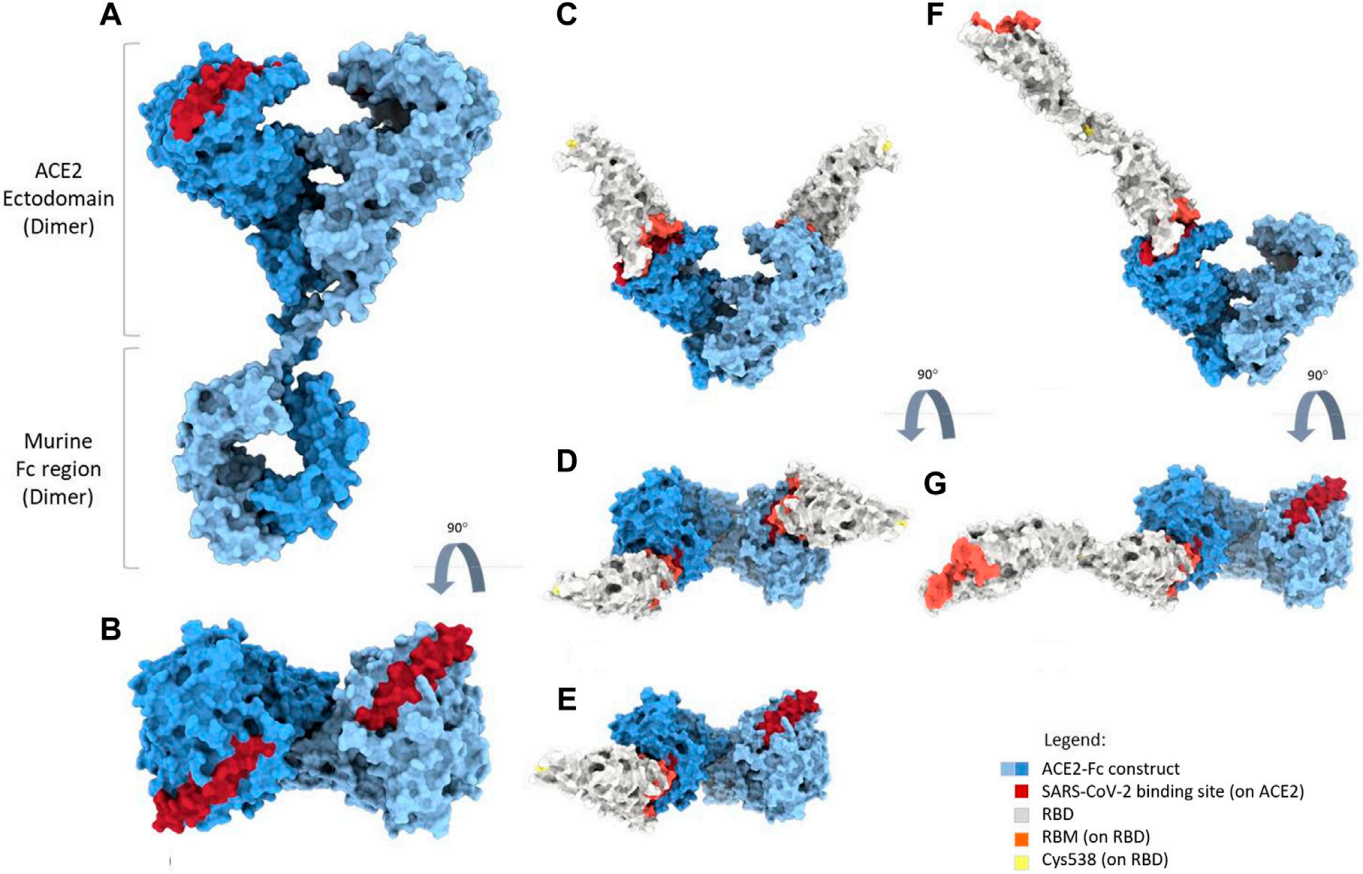
Three-dimensional models were generated from molecular dynamics experiments utilizing the amino acid sequences of two recombinant proteins previously studied using surface plasmon resonance (SPR): the mFc-ACE-2 chimeric protein and RBD (319-541)-His6 monomer and dimer. The figure shows the models. Part A shows the dimer structure for mFc-ACE-2, which includes ACE2 fused to the Fc region of mouse IgG1. The SARS-CoV-2 binding site on the ACE2 ectodomain is highlighted in dark red **(A,B)**. The middle panels display the RBD monomer interacting with ACE2. The RBD can occupy either one (part E) or both (parts **(C,D)**) binding sites. Cysteine 538 (C538) on the RBD, depicted in yellow, plays a crucial role in RBD dimerization. It lies opposite the receptor binding motif (RBM), shown in light red (parts **(C–E)**). For the RBD dimer, only one RBD molecule can interact with ACE2 at a time (parts **(F,G)**). Since the distance between the two ACE-2 binding sites is greater than 2.05Å (Sun et al., 2017; Wiedemann et al., 2020), one molecule of dimeric RBD cannot cover both sites, particularly when the sites have opposite orientations **(C,D)**. This causes steric interference, which hinders the bivalent binding of the RBD dimer. This experimental approach may clarify the outcomes of SPR experiments regarding the RBD and ACE2 interaction.

The SPR results suggest that the RBD dimer interacts with the chimeric mFc-ACE2 receptor similarly to the RBD monomer. Nevertheless, the data did not support a model where two RBD molecules bind to one ACE2 dimer simultaneously (results not shown). The RBD dimer did not saturate the binding sites, unlike the RBD monomer at the same concentration range, as shown by the shape of the curves in Supplementary Figure S9B. To fit the experimental data to a bivalent interaction model, either a low-affinity interaction or a lengthy dissociation time (e.g., hours or overnight) is required (Karlsson et al., 1995; Srinivas Reddy et al., 2011). In this case, the RBD-ACE2 affinity constants are in the nanomolar range, suggesting a high-affinity interaction (Shang et al., 2020). Moreover, the SPR experiments did not detect any bivalent interaction, as the dissociation time was barely 120 s. The three-dimensional molecular dynamics models, generated from the amino acid sequences of the ACE2 receptor and the RBD (319-541)-His6, further reinforce the notion that ACE2 cannot interact with both RBDs within the same dimeric RBD molecule (Figure 11). This is due to the limited distance between the two arms of the ACE2 receptor, where the SARS-CoV-2 binding sites are located (Figures 11A,B), which cannot accommodate both RBDs dimerized via the C538 residue.

ELISA confirmed the concentration-dependent binding of RBD (319-541)-His6 protein to ACE2, as shown in Supplementary Figure S10. At lower concentrations, the RBD dimer demonstrated greater sensitivity than the monomer, which can be attributed to the greater number of exposed epitopes. As a result, the detection antibody conjugate had a higher affinity for the dimer. This sensitivity discrepancy is not a result of an inequality in ACE2 binding affinity, as shown by the surface plasmon resonance outcomes.

Shortly, both the RBD monomer and dimer bind ACE2 strongly, but bivalent binding is not feasible.

### 3.19 Stability of purified RBD (319-541)-His6 monomer and dimer

Guidelines, such as the International Council for Harmonization’s Q1A (R2), recommend analyzing the changes in drug substance and product quality over time (ICH-Q1A, 2003). Currently, there are no reports available on RBD molecule shelf-life stability. We conducted a stability study on RBD (319-541)-His6 dimer and monomer to determine their shelf-life and proper storage conditions. This study examined the physical, chemical, and biological properties of the molecules over time to assess their stability.

The purified RBD monomer and dimer retained their integrity in solution at 4°C–8°C for at least 12 months, based on peptide mapping and glycosylation analysis. Furthermore, the biological activity of both forms, as measured by their ability to bind to the ACE2 receptor, remained intact throughout the storage period. These observations indicate that both the RBD monomer and dimer are stable under the tested conditions, which is promising for their applicability in vaccines. In-depth results can be found in Supplementary Tables S6, S7.

**FIGURE 12:**
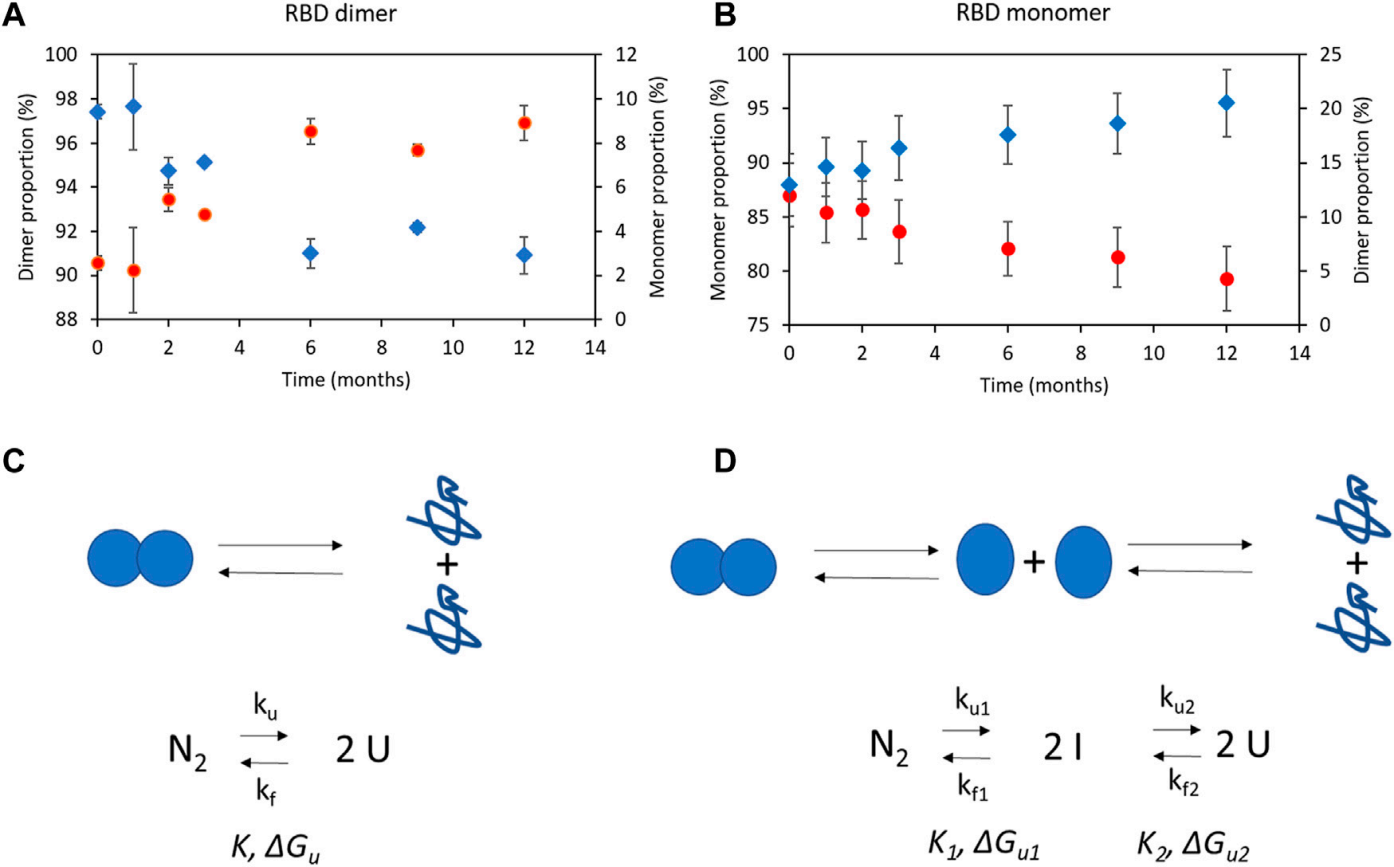
Proportion of purified RBD (319-541)-His6 species (monomer and dimer) measured by SEC-HPLC over time. The data represents the mean of three different samples, along with their standard deviation. **(A)** Purified RBD dimer with low RBD monomer levels. **(B)** Purified RBD monomer and dimer mixture. The proportion of RBD dimers is indicated by blue diamonds, while the proportion of RBD monomers is represented by red circles. Panel **(C)** illustrates a two-state transition, where the native state of the protein dimer (N2) can directly unfold into two unfolded protein monomers (U) with a first-order unfolding/dissociation rate constant (Ku). Panel **(D)** shows a three-state transition with an intermediate protein monomer (I). The native dimer state (N2) can unfold into an intermediate state (I) with a second-order folding rate constant (Kf), which then further unfolds into two unfolded monomers (U) with a first-order unfolding/dissociation rate constant (Ku). The equilibrium constant (K) and the Gibbs free energy of unfolding (ΔGu) are also depicted.

During the stability study, changes were observed in the proportion of RBD dimers and monomers, as determined by SEC-HPLC chromatograms (Figures 12A, B). Initially, the purified RBD dimer material contained 97.4% RBD dimer (blue symbols in Figure 12A) and 2.6% RBD monomer (red symbols in Figure 12A). After 6 months of storage at 4°C–8°C, the content of RBD dimers decreased to 91%, while the content of RBD monomers increased to 8.5%. In the purified mixture of RBD monomer and dimer, the original composition was 87% RBD monomer and 13% RBD dimer (Figure 12B). After 12 months of storage, the RBD monomer content decreased to 79.3%, while the RBD dimer content increased to 20.7%, indicating a conversion from the monomeric fraction to dimeric fraction during the storage period. It is worth noting that after the 12-month shelf stability study, the oligomerization peaks accounted for only 0.06% for RBD monomers (Supplementary Figure S11C) and 0.1% for RBD dimers (Supplementary Figure S11D), indicating that aggregation was not a significant concern during this period. Nevertheless, the purity of each RBD product met the established specifications: ≥40% monomeric form and ≤60% dimeric form for RBD monomer and ≥90% dimeric form for RBD dimer. The lower purity specification for the monomeric RBD is due to the production of the SOBERANA 02 vaccine antigen. This production involves the conjugation of RBD to tetanus toxoid, which requires the reduction of RBD to free all the C538 (Valdes-Balbin et al., 2021).

Aggregation mechanisms can diminish the abundance of protein monomers in favor of protein dimers or other oligomers (Kuzman et al., 2021), as observed in this study with RBD (319-541)-His6 (Figure 12B). Less commonly reported is the conversion of a protein homodimer into its monomeric form (Rumfeldt et al., 2008). One possible reason for the physical degradation of the RBD dimer to its RBD monomeric species during storage is protein dimer unfolding mechanisms. These mechanisms involve reversible reactions through two or three states, often resulting in two unfolded monomers with or without intermediate protein species (Neet and Timm, 1994). Figures 12C, D illustrate two mechanisms of protein dimer degradation based on the model described by Neet and Rumsfeldt, with the specific mechanism dependent on protein concentration (Neet and Timm, 1994; Rumfeldt et al., 2008). The behavior of the RBD dimer during the stability study aligns with this physical degradation process, where the monomer fraction increased while the dimer fraction decreased until reaching a steady state. This is likely due to the equilibrium between both RBD species.

Recapitulating, the RBD monomer and dimer retain their quality specifications in solution at 4°C–8°C for at least 12 months, with preserved biological activity.

### 3.20 Analysis of RBD (319-541)-His6 immunogenicity in mice

In mouse models, the immunogenicity of RBD vaccines has been used as an indicator of potential efficacy in humans. Studies conducted on BALB/c mice have demonstrated that RBD (319-541) vaccines, when administered with adjuvant Al(OH)_3_, can elicit an immune response at doses ranging from 0.1 to 30 µg (Dai et al., 2020; Dalvie et al., 2021; Pan et al., 2021).

**FIGURE 13:**
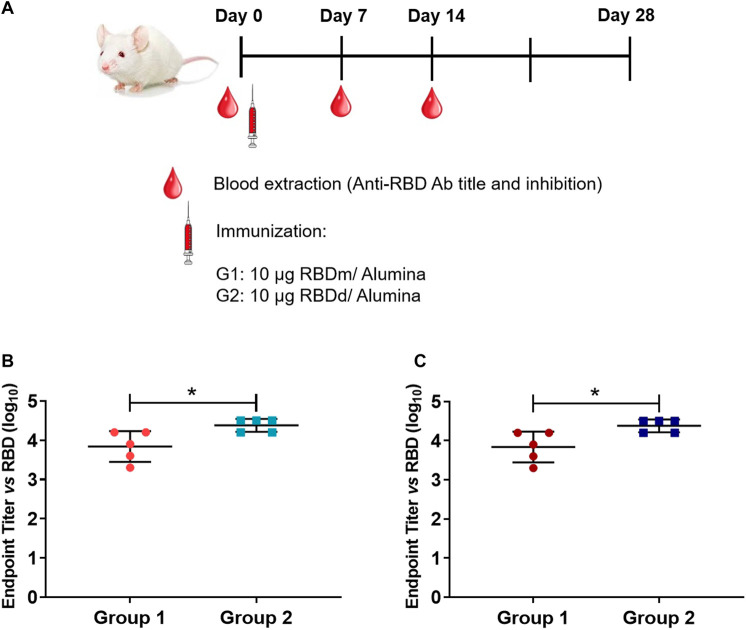
Two groups, G1 and G2, each consisting of five BALB/c mice each, were immunized with either the RBD monomer (represented by circles) or the RBD dimer (represented by squares), both absorbed in Al(OH)_3_ at a dosage of 10 µg (Panel **(A)**). On day 14, ELISA was performed to determine the IgG titers specific for RBD in the serum of the immunized mice (Panels **(B,C)**). Panel **(B)** shows the ELISA plates coated with the RBD monomer, while Panel **(C)** shows the plates coated with the RBD dimer. The graph indicates a significant statistical difference in Endpoint titers between the two groups (*p = 0.0159 according to the Mann-Whitney U test).

Figure 13 illustrates that 10 µg of RBD species (monomer or dimer) absorbed in Al(OH)_3_ and administered intramuscularly in BALB/c mice is immunogenic. No IgG responses were observed in any group on day 7. However, both the RBD monomer and dimer elicited strong antibody responses by day 14, with the dimer inducing higher IgG titers than the monomer. To ensure unbiased results, ELISA plates were coated with either RBD dimer or monomer during the assay. The geometric mean titer elicited by the RBD monomer was 6,964.4, which is lower than that obtained for the RBD dimer (24251.5). This finding is consistent with previous studies by Dai et al. (2020) and Dalvie et al. (2021), showing the superior immunogenicity of the RBD dimer in BALB/c mice.

## 4 Conclusion

In conclusion, we have developed a single process for producing RBD (319-541)-His6 monomer and dimer, adaptable to different COVID-19 vaccine platforms. Using CHO-K1 cells for RBD expression and selecting the best RBD-producing clones significantly contributes to production efficiency. To optimize RBD production, maintaining high cell culture viability and minimizing RBD exposure in the culture are key factors. Copper supplementation to the culture medium enhances RBD dimerization without affecting cell growth. Implementing perfusion cell culture with copper supplementation is an effective technique for generating both RBD forms, improving RBD dimer production. Purification can be carried out using conventional separation techniques such as IMAC, ion exchange chromatography, and size exclusion chromatography.

We have successfully demonstrated the scalability of our process by producing RBD antigens on a large-scale perfusion culture of 500 L. Large-scale production of RBD antigens can be facilitated by an on-site production platform allowing for high volumetric productivity in perfusion, as demonstrated in our study. Furthermore, a thorough examination of purified RBD antigens, including structural integrity, glycosylation patterns, stability, and immunogenicity, yields crucial information for guaranteeing the quality and effectiveness of the vaccine antigens. Future research could aim to quantitatively measure C538 disulfide scrambling levels and determine the relative abundance of distinct C538 sulfhydryl modifications.

Overall, our research offers significant insights into RBD production under various conditions and scales, which can help improve strategies for large-scale manufacturing of COVID-19 vaccines.

## Data Availability

The original contributions presented in the study are included in the article/Supplementary Material, further inquiries can be directed to the corresponding authors.
