# Supplementary material for: Development of a scalable single process for producing SARS-CoV-2 RBD monomer and dimer vaccine antigens
Source: Front Bioeng Biotechnol. 2023 Nov 17;11:1287551. doi: 10.3389/fbioe.2023.1287551 (PMC10693982; doi:10.3389/fbioe.2023.1287551)

Supplementary Material

1 Supplementary Materials and Methods

1.1 Further details for RBD (319-541)-His6 expression in CHO-K1 cells

The synthetic main fragment used to assemble the DNA sequence encoding the aa 328-533 segment of the SARS-CoV-2 spike protein was obtained from Eurofins (Germany). The Center for Genetic Engineering and Biotechnology (CIGB, Havana, Cuba) synthesized the required oligonucleotides for completing the gene encoding the R319-F541 sequence through overlapping PCR. Additionally, they produced the flanking oligonucleotides. The plasmid DNA was purified using the QIAprep Spin Miniprep Kit (Qiagen, Germany). The lentiviral vector pL6WBlast was obtained from CIGB (Cuba), and the pCMX-His intermediate vector was obtained from the Technical University of Braunschweig (Germany). Sequence verification of the genetic construct was performed by Microsynth-SEQLAB (Germany).

1.2 Further details for generating clones for RBD (319-541)-His6 production

To produce lentiviral particles carrying the RBDsint201 genetic construct, we transfected human embryonic kidney 293T (HEK-293T) cells (ATCC, CRL-3216) with the RBDsint201 genetic construct and auxiliary plasmids pLPI, pLPII, and LP/VSV-G (Invitrogen, USA) using 25 kDa linear polyethyleneimine (Polysciences, USA) as the transfecting agent. The mass ratio of the four plasmids used during transfection was 2:1:1:1. After 72 h of growth at 37°C in a 5% CO_2 atmosphere, we collected the resulting cell culture supernatant, and lentiviral particles were concentrated through precipitation with polyethylene glycol (Sigma Aldrich, USA). We measured the viral titers in the final preparation using the DAVIH Ag p24 ELISA kit (LISIDA, Cuba) according to the manufacturer's instructions, and defined 0.65% of the total virus concentration as infectious virus particles.

To transduce CHO-K1 cells (ATCC, CCL-61) with the lentiviral particles, we cultured the cells in 96-well plates at a density of 5000 cells in 200 µl/well using DMEM/F-12 (Dulbecco's Modified Eagle Medium/Nutrient Mixture F-12) supplemented with 5% fetal bovine serum (FBS) (HyClone, GE Healthcare, USA) for 24 h at 37 ºC in a 5% CO_2 atmosphere. Next, we added lentiviral particles to each well at a multiplicity of infection of 800, along with polybrene at 10 µg/ml, and incubated the cells for 8 h. To stop the infection, we added 10 µl of FBS to each well, and allowed the cells to recover overnight under the same conditions. We repeated this procedure twice every 24 h to increase the transduction efficiency. Cells from some wells were grown in DMEM/F-12 supplemented with 10% FBS and blasticidine at 2 µg/ml for 72 h after being transduced three times (referred to as 3x-transduced). After recovering for 72 h, 3x-transduced cells from some wells were subjected to three additional cycles of lentiviral infection every 24 h before being grown in supplemented medium as described above. These cells are referred to as 6x-transduced.

To select oligoclonal cell lines that produce high levels of biologically active RBD (319-541)-His6, we diluted the cells from each transduction well and grew them for 10 days across five 96-well plates in DMEM/F-12 supplemented with 10% FBS and blasticidine at 2 µg/ml. We then subjected the cell culture supernatants to ELISA using polyvinyl chloride microtiter plates coated with 10 µg/ml of recombinant ACE2/human Fc fusion protein, which captured the RBD. To detect the RBD, we used

anti-RBD monoclonal antibody (mAb) S1 (CIGB, Sancti Spiritus, Cuba) and anti-mouse IgG antibody conjugated to horseradish peroxidase. The oligoclonal cell lines that displayed the highest ELISA signals (absorbance at 490 nm) were selected and transferred to 24-well plates for further expansion, freezing, adaptation to growth in suspension in the absence of FBS, and initial expression studies.

To ensure the production of consistent results, we cloned the same cell lines in parallel through limiting dilution in 96-well plates at a density of 0.8 cells/well in DMEM/F-12 supplemented with 5% FBS and blasticidine at 2 µg/ml. After two weeks, we tested the supernatants of wells showing cell growth (presumably clonal) using ELISA as previously described. The clones that exhibited the highest secretion of RBD, as determined by ELISA, were expanded, frozen, and adapted for growth in suspension. To further evaluate their potential, we selected a panel of ten clones from each lentiviral transduction protocol (three rounds of transduction and six transduction cycles) for small-scale (7 ml) expression studies in shaking flasks (25 cm^2) (Greiner Bio-One GmbH, Germany).

1.3 Further details for small-scale purification of RBD

For IMAC chromatography, the Chelating Sepharose Fast Flow XL column was equilibrated with 20 mM sodium phosphate buffer (pH 7.4) containing 300 mM NaCl, 5 mM imidazole, and 0.1% Tween 20. After equilibration, the column was washed with the same buffer solution, except the imidazole concentration was increased to 10 mM. The RBD was then eluted with PBS (pH 9) containing 200 mM imidazole and 0.1% Tween 20. All IMAC steps were performed at a flow rate of 300 cm/h.

For buffer exchange, the Sephadex G-25 column was equilibrated with 50 mM sodium phosphate buffer (pH 6) containing 30 mM NaCl and operated at a flow rate of 150 cm/h. A volume of RBD-containing intermediate product corresponding to 20% of the column volume was loaded onto the Sephadex G-25 column.

For cation exchange chromatography, the SP Sepharose Fast Flow column was pre-equilibrated with 50 mM sodium phosphate buffer (pH 6) containing 30 mM NaCl. The flow rate was 150-200 cm/h. The bound RBD species were eluted using 50 mM sodium phosphate buffer (pH 6) containing 200 mM NaCl.

For SEC-HPLC, the Superdex 200 pregrade column was pre-equilibrated with PBS and eluted at a flow rate of 30 cm/h.

2 Supplementary Figures and Tables

2.1 Supplementary Figures

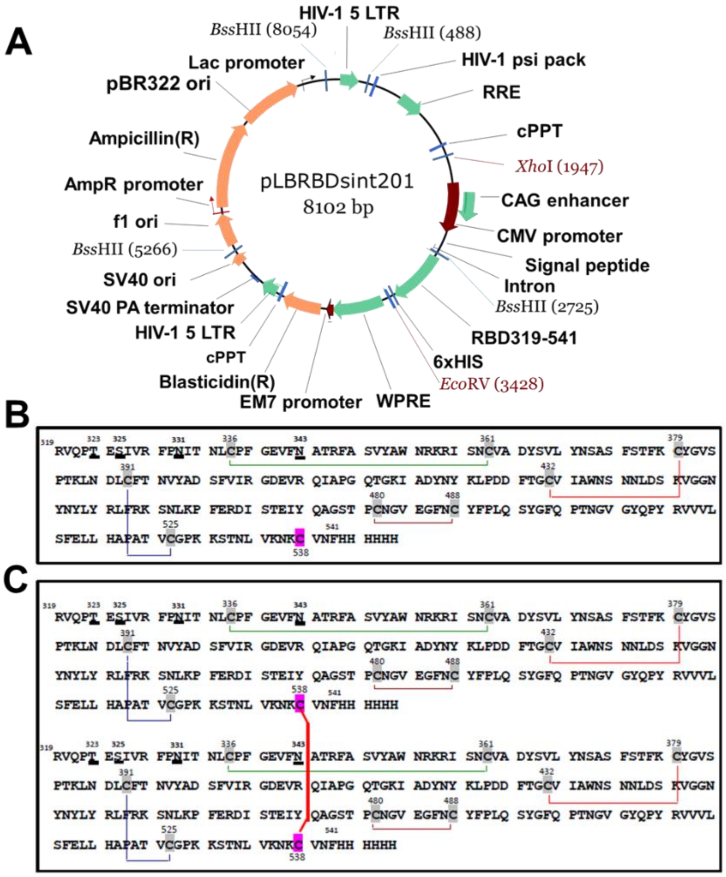

Supplementary Figure S1. (A) Schematic representation of the lentiviral plasmid pLBRBDsint201, which was used for stable transduction of CHO-K1 cells. The expression cassette transferred from an intermediate vector into the pL6WBlast scaffold contains the CMV promoter, the gene encoding a signal peptide of the mouse heavy chain variable region (including one intron), the gene encoding RBD (319-541), and a sequence encoding a C-terminal hexahistine tag. The genetic construct also contains a WPRE sequence for enhanced expression, a blasticidin resistance gene as a selection marker for transduced cells, the SV40 terminator, an ampicillin resistance gene for plasmid amplification in bacteria, and other typical lentiviral factors. Panel B shows the expected amino acid sequence of the RBD monomer, while panel C shows the amino acid sequence of the RBD dimer. The thinner lines represent the four intrachain disulfide bonds (C336-C361, C379-C432, C480-C488, and C391-C525). Notably, C538 is present in the molecules as a free or modified cysteine and as a homodimer (C538-C538). Residues marked with subscripts N and T/S correspond to potential N- and O-glycosylation sites.

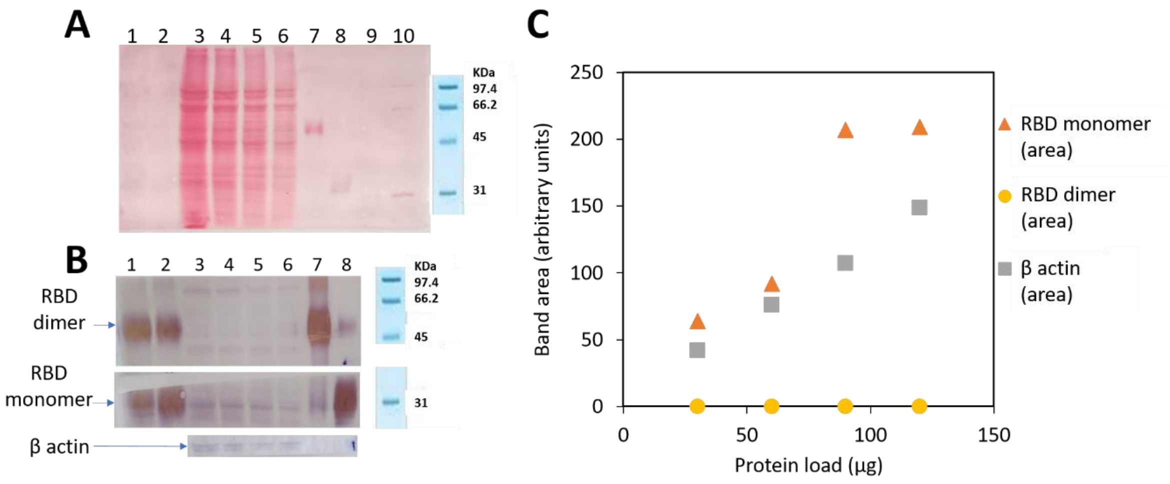

Supplementary Figure S2. On the formation of the RBD (319-541)-His6 dimer assembly. (A) Protein transfer from gel to membrane was confirmed by Ponceau red staining, which visualizes the total protein load. (B) Western blot was revealed with ACE2-Fc and anti-human (γ-chain specific)-HRP. Lane 1, CHO-K1 cell culture supernatant with 50 µM $CuSO_4$; lane 2, same as lane 1 but without $CuSO_4$; lanes 3-6, decreasing amounts (120, 90, 60 and 30 µg) of total intracellular proteins; lane 7, RBD dimer control; lane 8, RBD monomer control; lane 9, blank; lane 10, molecular mass marker. (C) Densitometric analysis of monomeric and dimeric RBD (as detected by Western blot) and β-actin, each relative to total protein in lanes 3-6. The densitometric analysis showed that there was agreement between the area corresponding to β-actin bands and the area corresponding to total protein loading per lane. A similar behavior was observed for the RBD monomer. Despite the increasing amount of intracellular protein loading in the lanes, an RBD dimer band was not detected in any of the samples.

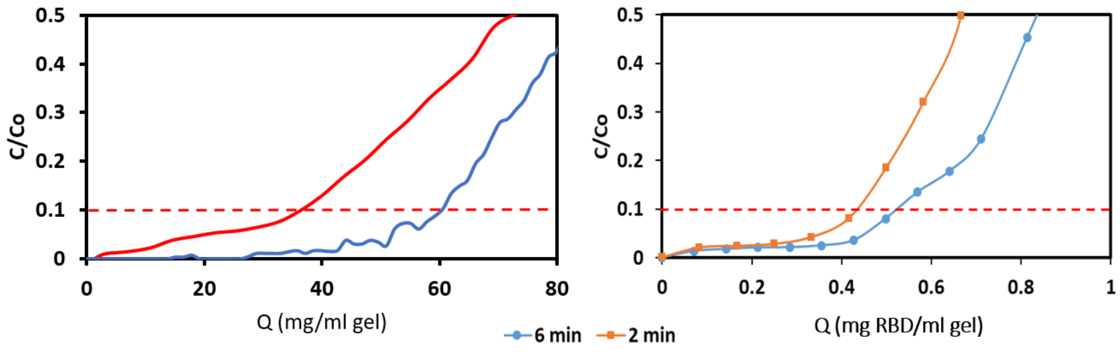

Supplementary Figure S3. Breakthrough curve for RBD adsorption on IMAC using purified RBD (328-533)-His6 (on the left panel) and supernatant from 39-3x culture (on the right panel) at two different residence times (red, 2 min; blue, 6 min). Column, XK16/20 packed with 30 ml of Chelating Sepharose Fast Flow. Equilibration buffer, 20 mM sodium phosphate, 500 mM NaCl, and 5 mM imidazole, pH 7.4. The dynamic binding capacity of Ni(II)-loaded IMAC gel matrix was found to be 51 mg/ml (at 2 min) and 58 mg/ml (at 6 min) when purified RBD was used as the sample to load the column. However, when the cell culture supernatant containing RBD was used as the sample, the dynamic binding capacity was reduced by 100-fold to 0.43 mg/ml (at 2 min) and 0.55 mg/ml (at 6 min).

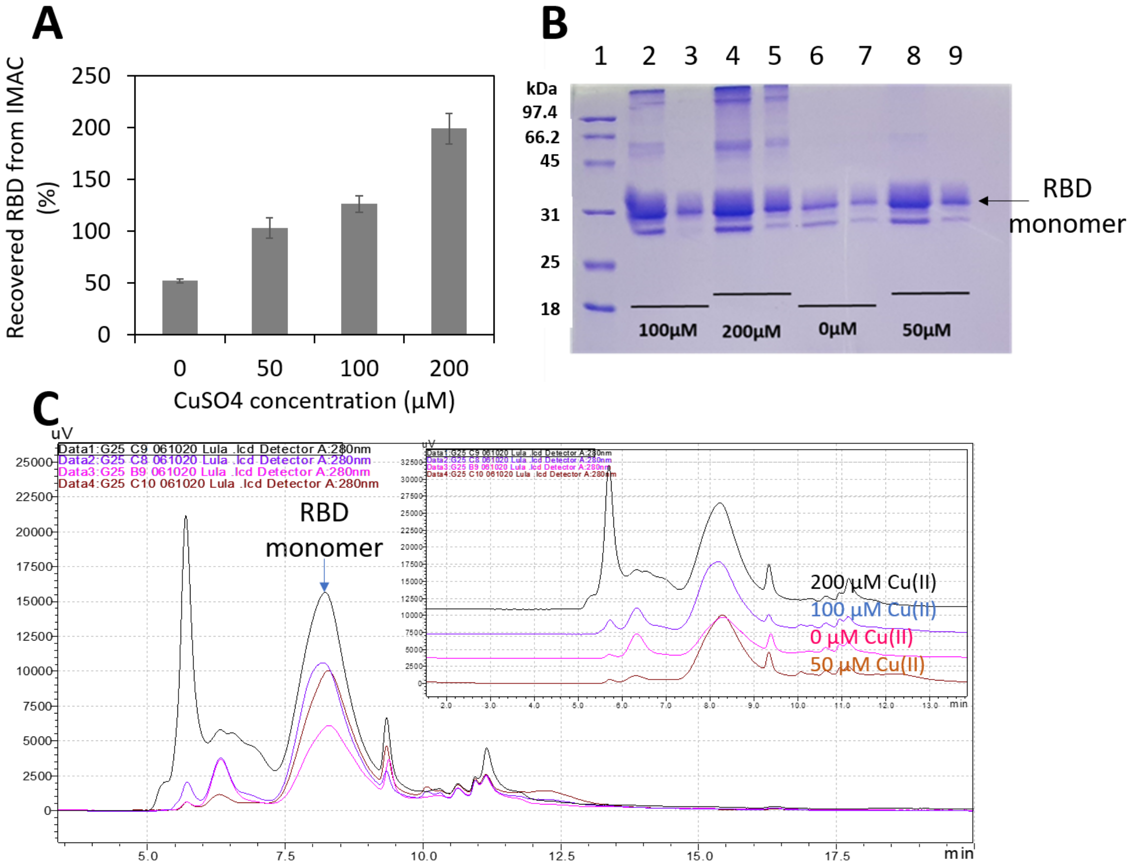

Supplementary Figure S4. Effect of adding copper sulfate to clarified cell supernatant containing RBD (328-533)-His6 on subsequent IMAC separation. (A) Yield of RBD eluted from the IMAC column after application of monomeric RBD in cell supernatant supplemented with different concentrations of copper sulfate (50, 100 and 200 µM). It is worth noting that 50 µM of $CuSO_4$ is the concentration used in the culture medium during the culture of the CHO cells producing RBD (319-541)-His6. Notably, the addition of copper sulfate resulted in an increase in RBD recovery, indicating that this approach can enhance the efficiency of the IMAC separation process. (B) SDS-PAGE of IMAC elution samples (lanes 2, 4, 6 and 8) and G25-mediated buffer exchange (lanes 3, 5, 7 and 9). The copper sulfate concentration for each experimental condition is shown at the bottom of the figure. C) SEC-HPLC of IMAC elution samples with copper at different concentrations. SDS-PAGE analysis (lanes 2, 4, and 5) and SEC-HPLC profiling revealed that when copper sulfate concentrations exceeded 50 µM, larger proteins with a molecular mass of over 97 000 Mr were eluted from the IMAC gel matrix. This could be attributed to the capture of highly oxidized and aggregated RBD species or other non-specific protein species present in the original loaded material. These findings support the use of 50 µM as the appropriate molarity of copper sulfate for conditioning the cell culture supernatant containing RBD prior to IMAC purification, ensuring that larger, unwanted protein species are not eluted and recovered during the purification process. In fact, using 50 µM copper sulfate, we were able to obtain RBD with a higher purity level (76.87%) compared to when the material was adjusted to concentrations of 100 µM (53.24%) or 200 µM (44.09%) of copper sulfate (data not shown).

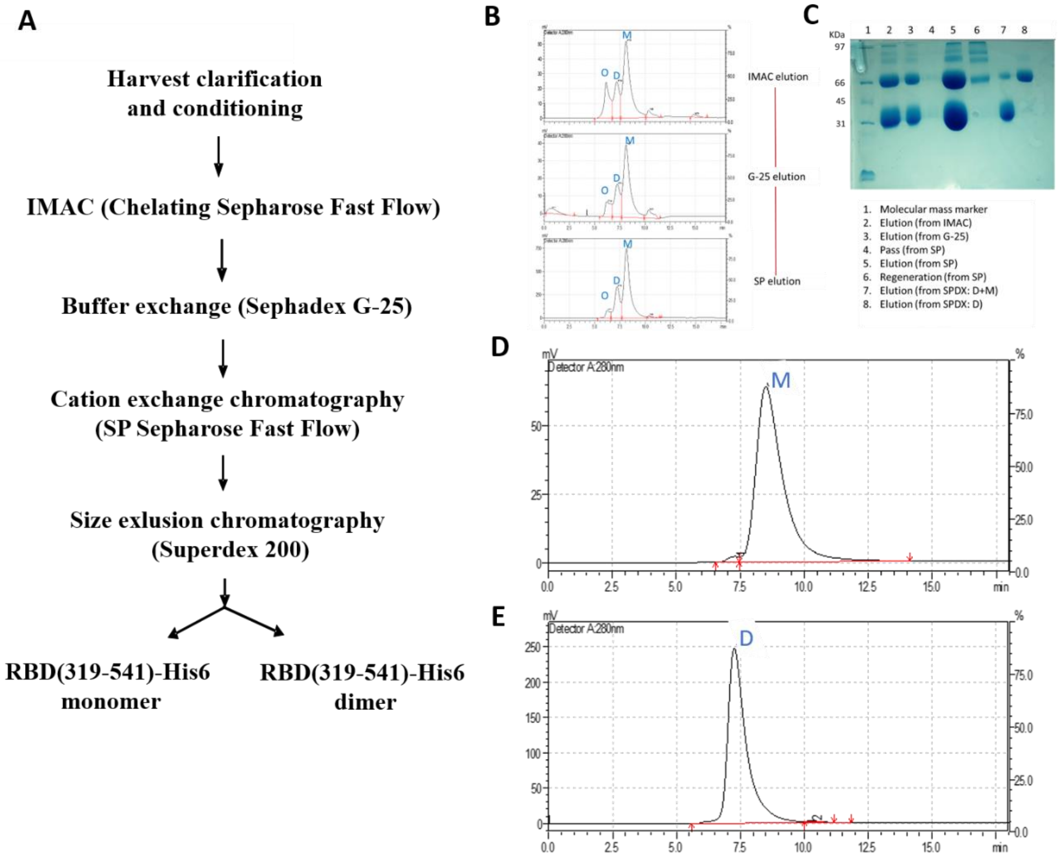

Supplementary Figure S5. Purification of RBD (319-541)-His6 from culture supernatant (clone 39-3x). (A) RBD-containing culture supernatant was conditioned by adding 50 µM $CuSO_4$ and subjected to IMAC chromatography. This was followed by a desalting step with Sephadex G25 and then SP cation exchange chromatography. The purification samples were analyzed by analytical SEC-HPLC (B) and SDS-PAGE (C). After cation exchange chromatography, the species of interest (RBD monomer [D] and RBD dimer [E]) were separated by SEC chromatography. O, oligomer; D, dimer; M, monomer.

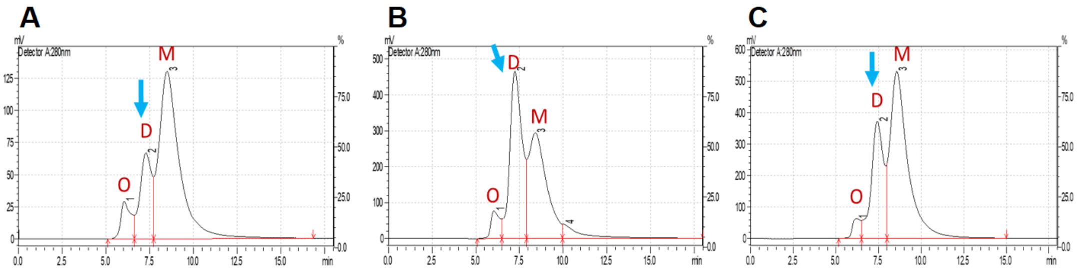

Supplementary Figure S6. Proportion of RBD (319-541)-His6 oligomers, dimers, and monomers prior to the final purification step. Size exclusion-HPLC profile of RBD-containing clarified supernatant separated by cation exchange from three different stages of the perfusion process: A, stage 1; B, stage 2; and C, stage 3. The materials from stage 1 and 3 were free of copper ions. O, peak of RBD oligomers; D, peak of RBD dimers; M, peak of RBD monomers.

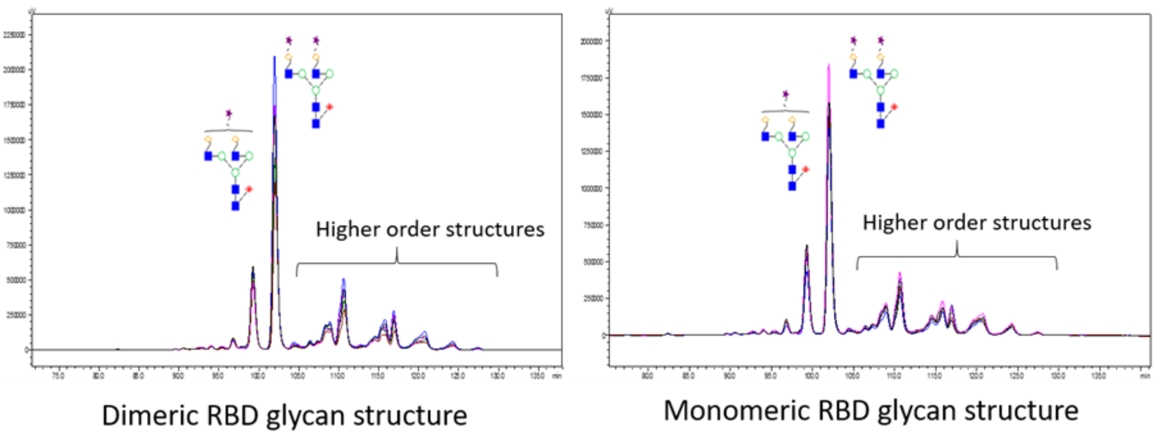

Supplementary Figure S7. Normal-phase HPLC chromatograms of the glycosidic structures of dimeric (A) and monomeric (B) RBD (319-541)-His6. Each panel shows the overlay of chromatograms from three batches of RBD, with lines in blue, black, and pink. The main glycan species are represented by symbols such as blue squares for N-acetylglucosamine, empty circles for mannose, red diamond for fucose, yellow diamond for galactose, and purple star for sialic acid. The figure suggests that the glycosylation patterns of dimeric and monomeric RBD are similar, with several major peaks observed in both chromatograms.

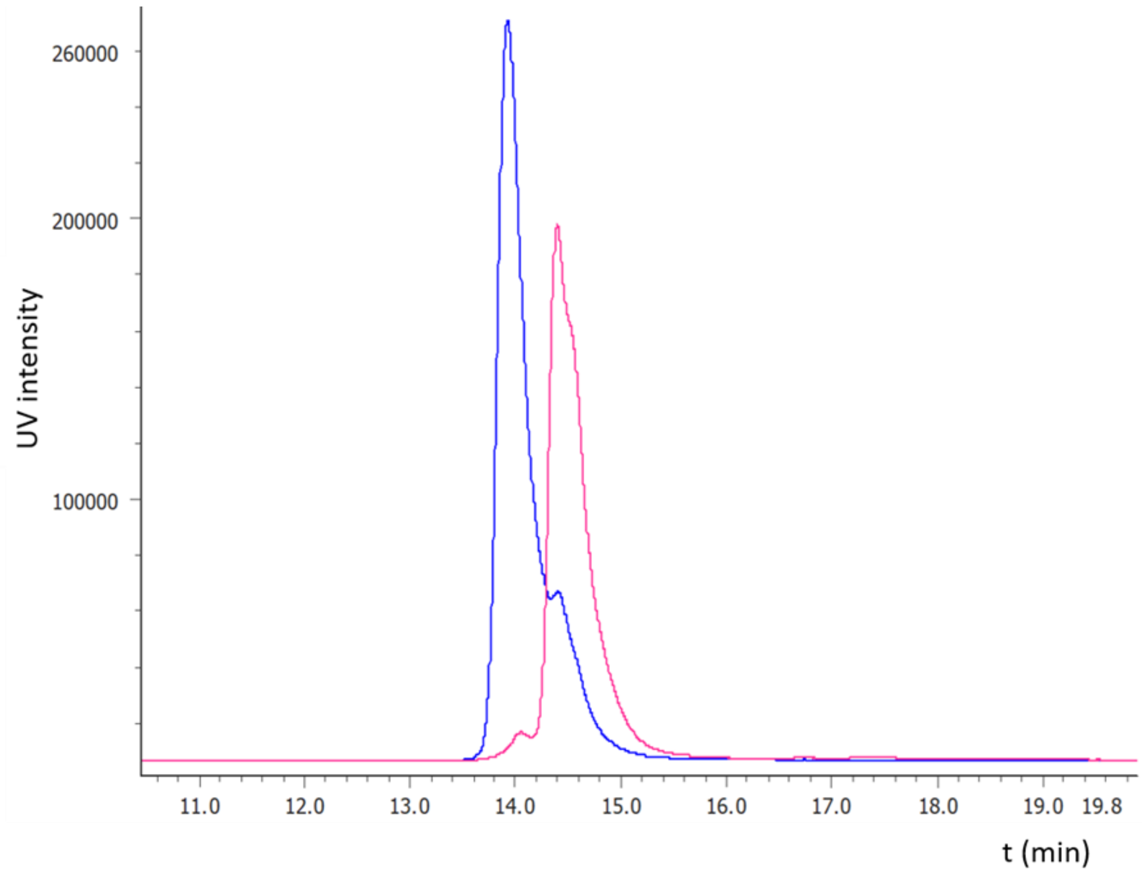

Supplementary Figure S8. RP-HPLC separation of monomeric RBD (319-541)-His6 represented in blue and dimeric RBD in red. Chromatography was performed on an Aeris 3.6 µm Widepore C4 column at 1 ml/min, with an acetonitrile gradient in TFA from 0.5% to 100% in 25 min.

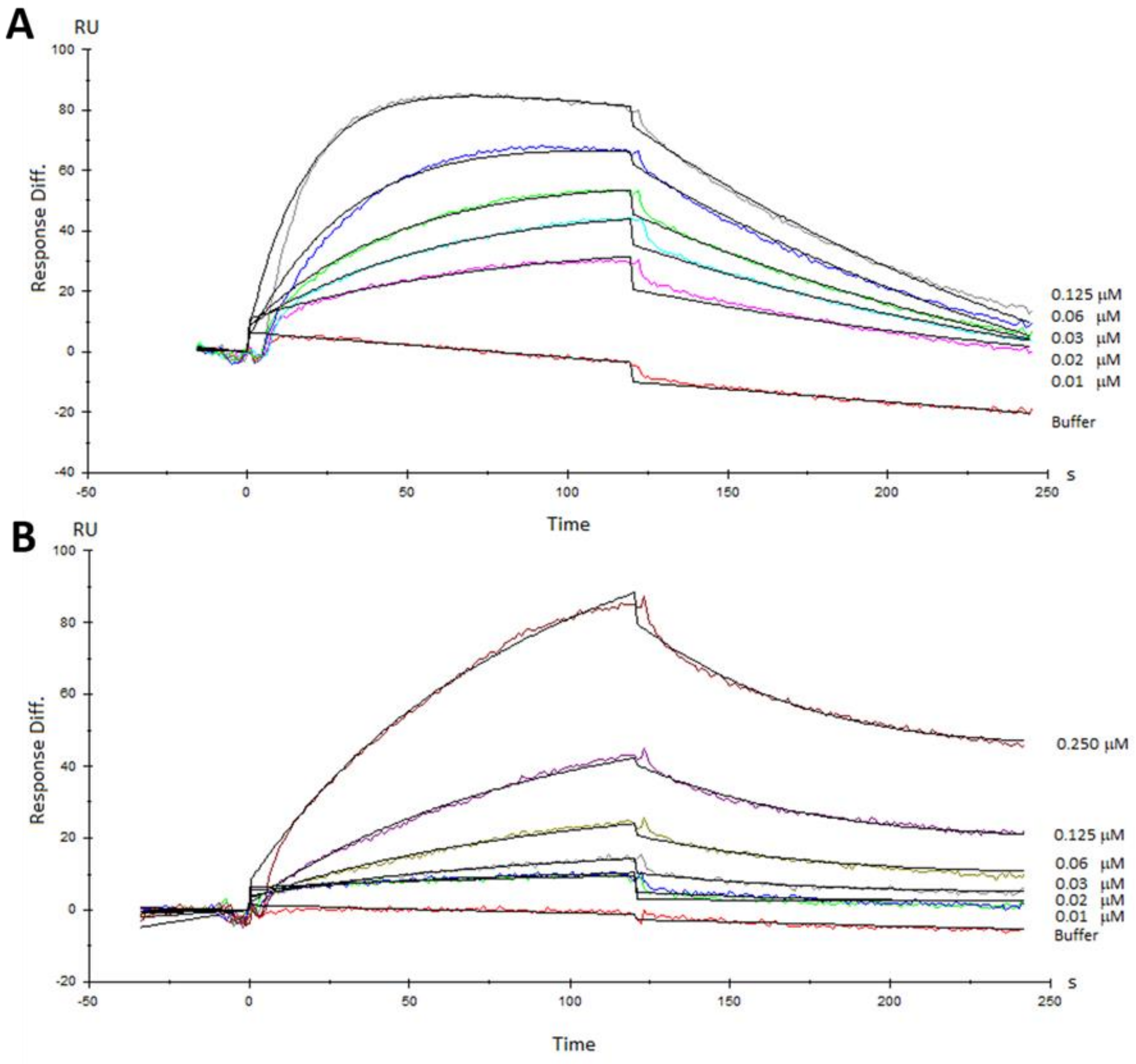

Supplementary Figure S9. Surface plasmon resonance (SPR) of RBD (319-541)-His6 species interacting with the ACE2 receptor immobilized on a protein A sensor chip. (A) SPR sensorgrams showing one replicate experiment of the RBD monomer dissolved in phosphate-buffered saline (PBS), pH 7.2. (B) SPR sensorgrams showing one replicate of the RBD dimer in PBS, pH 7.2. Each colored curve represents the indicated protein concentration on the right side. The black curves show the fit of the data to a 1:1 Langmuir binding model, yielding chi-squared values below 1.66.

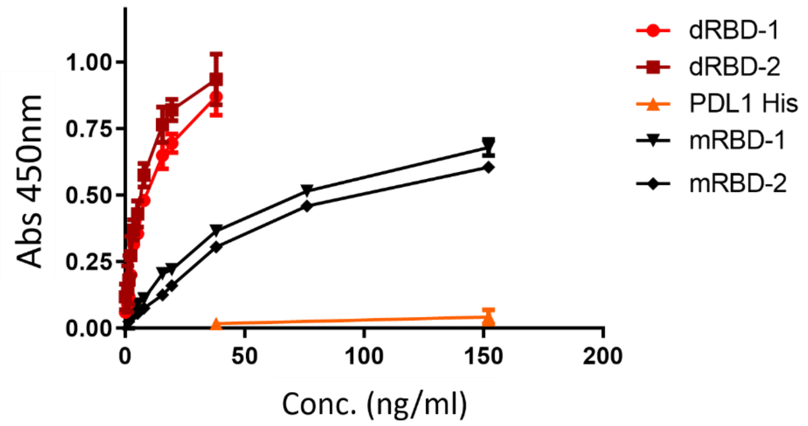

Supplementary Figure S10. Recognition of RBD (319-541)-His6 by the ACE2 receptor. Plates were coated with 200 ng/well of human ACE-2-Fc, blocked, and then loaded with samples. Mouse anti-RBD antibody and horseradish peroxidase-conjugated anti-mouse antibody were added for RBD detection. The data shown represent the mean ± standard deviation of absorbance at 450 nm from triplicate wells. dRBD, RBD (319-541)-His6 dimer; mRBD, RBD (319-541)-His6 monomer; PDL1, programmed death ligand 1.

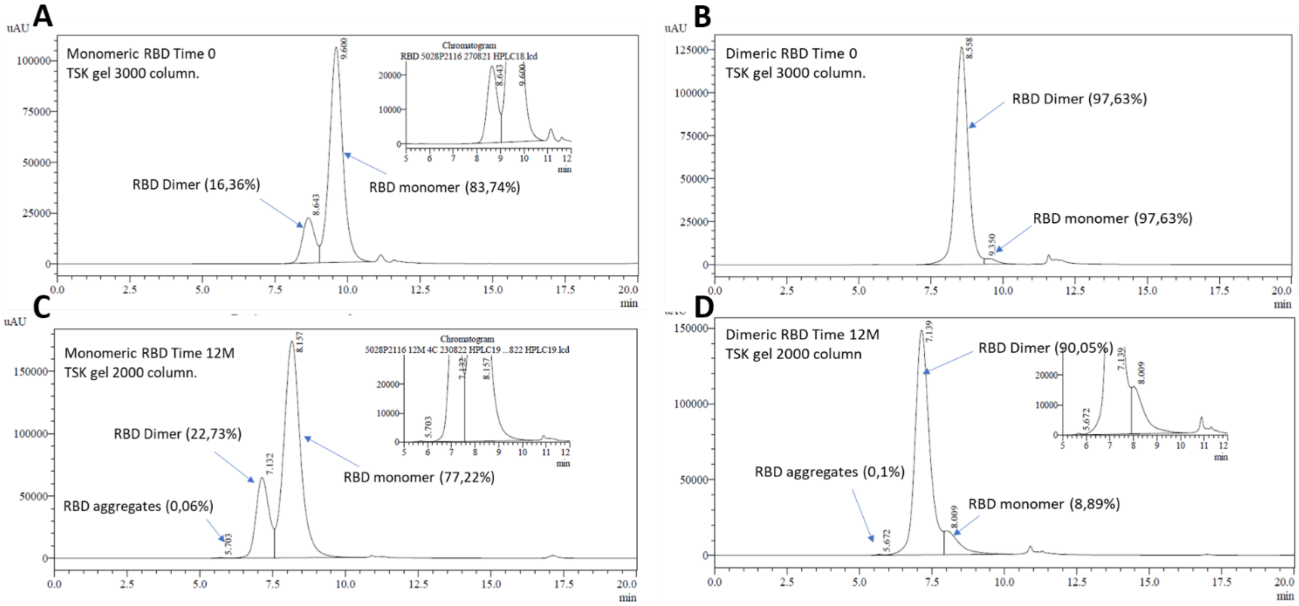

Supplementary Figure S11. Representative SEC-HPLC chromatograms of RBD monomer and dimer during shelf-life stability studies. The figure showcases the chromatographic profiles of monomeric RBD (A) and dimeric RBD (B) at the beginning of stability study. For separation, RBD-containing samples were loaded onto a TSK gel 3000 column. Additionally, the chromatographic profiles of monomeric RBD (C) and dimeric RBD (D) after 12 months of storage at 4°C are displayed. In this case, the RBD-containing samples were loaded onto a TSK gel 2000 column. The fractions corresponding to monomeric RBD, dimeric RBD, and RBD aggregates, along with their respective percentages, are indicated by arrows in the figure.

2.2 Supplementary Tables

Supplementary Table S1. Mean values of relevant variables characterizing each stage of the perfusion process described in Fig. 5 (primary text). Xv, concentration of viable cells; D, dilution rate; CSPR, cell-specific perfusion rate; qRBD, RBD (319-541)-His6 specific production rate; qGlc specific glucosa uptake rate; qO2, specific oxygen uptake rate; VP, volumetric productivity; dRBD, RBD (319-541)-His6 dimer; d, days. The values are averaged over six to eight replicates, and the standard deviations are also shown.

	Run time	Copper	Xv (cel/ml)	D (vvd)	CSPR (nl/cel/d)	RBD (mg/l)	qRBD (pcd)	qGlc (mmol/cel/h)	qO2 (mmol/cel/d)	VP (mg/l/d)	dRBD (%)
**Stage 1**	9 d	-	(8±2) x 10^6	1.2 ± 0.5	0.17 ± 0.05	23 ± 6	4.1 ± 0.8	(1±1) x 10^{-10}	(5±1) x 10^{-10}	23 ±5	23
**Stage 2**	5 d	+	(12±1) x 10^6	1.59 ± 0.05	0.134 ± 0.008	70 ± 14	9 ± 1	(5.34 ± 0.08) x 10^{-10}	(7.3±0.9) x 10^{-10}	74 ± 11	51
**Stage 3**	6 d	-	(12±1) x 10^6	1.59 ± 0.08	0.12 ± 0.01	78 ± 5	10 ± 1	(5.5±0.1) x 10^{-10}	(1.2±0.2) x 10^{-10}	78 ± 5	28

Supplementary Table S2. ESI-MS spectral peak assignments for independent batches of trypsin-digested RBD (319-541)-His6. To facilitate identification, RBD samples were treated with N-ethylmaleimide to ensure detection of native S-S bonds. The samples were also N-deglycosylated to improve the efficiency of proteolytic digestion and confirm the sequence of protein regions containing N-glycosylation. Tryptic digestion peptides were assigned based on agreement between theoretical and experimental monoisotopic m/z values. Nd, not determined.

Row	Code	Theor *m/z*	z	*Experimental m/z* (Monomer)			*Experimental m/z* (Dimer)			Description
				5028/ P2109	5028/ P2110	5028/ P2111	5016/ P2116	5016/ P2117	5016/ P2118	
1	R319-R328	592.84	2	592.85	nd	592.85	nd	592.81	nd	319RVQPTESIVR328 (N-terminal, non-glycosylated)
2	V320-R328	514.79	2	514.80	514.77	514.80	514.79	514.76	514.78	320VQPTESIVR328 (T323/S325 non-glycosylated)
3	F347-R355	557.28<br>1113.55	2<br>2	557.28<br>1113.54	557.28<br>1113.52	557.29<br>1113.55	557.28<br>1113.54	557.25<br>1113.47	557.29<br>1113.52	347FASVYAWNR355
4	F347-K356	414.55	3	414.55	414.56	414.57	414.56	414.51	414.56	347FASVYAWNRK356
5	K356-R357	303.21	1	303.21	303.22	303.22	303.22	303.20	303.22	356KR357
6	G404-R408	575.28	1	575.27	575.27	575.28	575.28	575.24	575.27	404GDEVR408
7	Q409-K417	899.50<br>450.25	1<br>2	899.49<br>450.26	899.49<br>450.26	899.51<br>450.27	899.49<br>450.25	899.49<br>450.23	899.50<br>450.26	409QIAPGQTGK417
8	I418-K424	886.43<br>443.72	1<br>2	886.43<br>443.72	886.42<br>443.72	886.44<br>443.73	886.43<br>443.72	886.38<br>443.70	886.43<br>443.72	418IADYNYK424
9	V445-R454	1218.59<br>609.80	1<br>2	1218.60<br>609.81	1218.53<br>609.80	1218.60<br>609.81	1218.55<br>609.80	1218.48<br>609.77	1218.56<br>609.80	445VGGNYNYLYR454
10	L455-R457	435.27	1	435.27	435.27	435.29	435.28	435.24	435.27	455LFR457
11	S459-R466	495.77	2	495.78	495.77	495.79	495.78	495.75	495.77	459SNLKPFER466
12	G496-R509	792.38	2	792.38	792.34	792.41	792.38	792.34	792.36	496GFQPTNGVGYQPYR509
13	K529-K535	395.25	2	395.25	395.25	395.26	395.25	395.23	395.25	529KSTNLVK535
14	S530-K535	661.39<br>331.20	1<br>2	661.40<br>331.22	661.40<br>331.21	661.40<br>331.21	661.41<br>nd	661.35<br>nd	661.38<br>nd	530STNLVK535
15	N536-K537	261.16	1	261.15	261.16	261.16	261.16	261.14	261.15	536NK537
16	S-S538-391	823.63<br>659.11	4<br>5	823.63<br>659.10	823.64<br>659.12	823.65<br>659.13	823.64<br>659.11	823.60<br>nd	823.65<br>659.10	538CVNF541-*HHHHHH*<br>| 387LNDLCFTNVYADSFVIR403<br>**(C538-C391)**

17	C538+NEM	477.21	3	477.22	477.22	477.22	477.21	477.18	477.21	538**CNEM**VNF541-*HHHHHH* **(C538+NEM, +125 Da, C-terminal)**
18	C538 - 49 Da	419.19	3	419.19	419.20	419.20	419.17	419.20	419.22	538C*VNF541-*HHHHHH* **(C538 - 49 Da, -[S+NH3], C-terminal)**
19	C538 - 34 Da	424.20	3	424.23	424.20	424.22	424.23	424.18	424.22	538C*VNF541-*HHHHHH* **(C538 - 34 Da, Cys dehydroalanine, C-terminal)**
20	C538 SH	435.53	3	435.53	435.52	435.54	435.53	nd	nd	538CVNF541-*HHHHHH* **(C538 free (SH) unmodified, C-terminal)**
21	C538 + 32 Da	446.18/	3	446.19	446.19	446.20	nd	nd	nd	538C*VNF541-*HHHHHH* **(C538 + 32 Da, Cys + [S] or [O_2], C-terminal)**
22	C538 + 32 Da	446.19		-	-	-	-	-	-	538C*VNF541-*HHHHHH* **(C538 + 32 Da, Cys + [S] or [O_2], C-terminal)**
23	C538 + 64 Da	456.85	3	nd	456.85	456.86	nd	nd	nd	538C*VNF541-*HHHHHH* **(C538 + 64 Da, Cys + [SO_2], C-terminal)**
24	C538+86 Da	-	3	464.27	464.25	464.25	nd	nd	nd	538C*VNF541-*HHHHHH* **(C538 +86 Da, C-terminal)**
25	C538+90 Da	-	3	465.53	465.52	465.54	nd	nd	nd	538C*VNF541-*HHHHHH* **(C538 +90 Da, C-terminal)**
26	C538+Cys	475.19	3	475.20	475.20	475.20	475.19	475.21	475.21	538CVNF541-*HHHHHH*<br>| NH2-**C**-COOH<br>**(Cys+C538, +119 Da, C-terminal)**
27	C538+ hCys	479.87	3	479.86	479.87	nd	nd	nd	nd	538CVNF541-*HHHHHH*<br>| NH2-homo**C**-COOH<br>**(homoCys+C538, +133 Da, C-terminal)**
28	C538+778 Da	-	4<br>3	nd<br>nd	nd<br>nd	480.97<br>640.96	480.96<br>640.94	480.93<br>640.90	480.96<br>640.94	538C*VNF541-*HHHHHH* **(C538 +778 Da, C-terminal)**
29	C538+150 Da	-	3	485.55	485.54	485.55	nd	nd	485.54	538C*VNF541-*HHHHHH* **(C538 +150 Da, C-terminal)**
30	C538+151 Da	-	3	485.87	485.87	nd	nd	nd	nd	538C*VNF541-*HHHHHH* **(C538 +151 Da, C-terminal)**
31	C538+161 Da	-	3	489.21	489.22	489.19	nd	nd	nd	538C*VNF541-*HHHHHH* **(C538 +161 Da, C-terminal)**
32	C538+167 Da	-	3	491.22	491.22	491.22	nd	nd	491.22	538C*VNF541-*HHHHHH* **(C538 +167 Da, C-terminal)**
33	C538+CG	494.20	3	494.20	494.21	494.21	nd	nd	nd	538CVNF541-*HHHHHH*<br>| NH2-CG-COOH<br>**(C538 + truncated glutathione, +176Da, C-terminal)**

34	C538+191 Da	-	3	499.20	499.20	499.22	nd	nd	nd	538C*VNF541-*HHHHHH* **(C538 +191 Da, C-terminal)**
35	C538+205 Da	-	3	503.87	503.87	503.87	nd	nd	nd	538C*VNF541-*HHHHHH* **(C538 +205 Da, C-terminal)**
36	C538+EC	518.21	3	518.23	518.23	518.22	nd	nd	nd	538CVNF541-*HHHHHH*<br>| NH2-EC-COOH<br>**(C538 +truncated glutathione, +248Da, C-terminal)**
37	C538+262 Da	-	3	522.89	522.89	522.89	nd	nd	nd	538C*VNF541-*HHHHHH* **(C538 +262 Da, C-terminal)**
38	C538+272 Da	-	3	526.21	526.22	526.21	nd	nd	nd	538C*VNF541-*HHHHHH* **(C538 +272 Da, C-terminal)**
39	C538+ECG	537.21	3	537.22	537.22	537.22	nd	nd	nd	538CVNF541-*HHHHHH*<br>| NH2-ECG-COOH<br>**(C538 glutathione, +305 Da, C-terminal)**
40	C538+430 Da	-	3	578.90	578.89	578.91	nd	nd	nd	538C*VNF541-*HHHHHH* **(C538 +430 Da, C-terminal)**
41	C538<br>+C≡N	443.86<br>665.28	3<br>2	443.85<br>nd	443.86<br>665.29	443.87<br>665.32	443.86<br>665.31	443.84<br>665.24	443.86<br>665.30	538CVNF541-*HHHHHH*<br>| C≡N<br>**(C538 cyanated, +25 Da, C-terminal)**

Supplementary Table S3. Detection of RBD (319-541)-His6 disulfide bonds by ESI-MS, in six independent 500-liter batches. The "code" column refers to the cysteine residues forming the disulfide bond, and the "theor m/z" column refers to the theoretical mass-to-charge ratio below which the experimental value was found for each batch of RBD. Nd, not determined.

Row	Code	Theor *m/z*	z	*Experimental m/z* (Monomer)			*Experimental m/z* (Dimer)			Description
				**5028/ P2109**	**5028/ P2110**	**5028/ P2111**	**5016/ P2116**	**5016/ P2117**	**5016/ P2118**	
1	S-S 336-361	1452.35<br>1089.51	3<br>4	1452.34<br>1089.53	1452.30<br>1089.53	1452.37<br>1089.54	1452.36<br>1089.53	nd<br>1089.47	1452.34<br>1089.51	329FP **D** **ITNLC**PFGEVF **D** ATR346<br>| 358ISNCVADYSVLYNSASFSTFK378<br>**(C336-C361, N331/343 D)**
2	S-S 379-432	1020.81<br>765.86	3<br>4	1020.82<br>765.87	1020.82<br>765.87	1020.84<br>765.88	1020.81<br>765.87	1020.75<br>765.82	1020.81<br>765.87	379CYGVSPTK386<br>| 425LPDDFTGCVIAWNSNNLDSK444<br>**(C379-C432)**
3	S-S 379-432	786.37<br>982.71	5<br>4	786.38<br>982.74	786.39<br>982.70	786.36<br>982.76	nd<br>nd	nd<br>nd	nd<br>nd	379CYGVSPTK386<br>| 418IADYNYKLPDDFTGCVIAWNSNNLDSK444<br>**(C379-C432+418IADYNYK424, 1 incomplete fragment)**
4	S-S 391-525	992.52<br>794.22	4<br>5	992.50<br>794.23	ND<br>ND	ND<br>ND	992.51<br>794.21	nd<br>nd	nd<br>nd	387LNDLCFTNVYADSFVIR403<br>| 510VVVLSFELLHAPATVCGPK528<br>**(C391-C525)**
5	S-S 480-488	1589.38<br>1192.29	3<br>4	1589.40<br>1192.27	1589.41<br>1192.29	1589.37<br>1192.31	1589.41<br>1192.30	1589.30<br>1192.25	1589.37<br>1192.27	467DISTEIYQAGSTPCNGVEGFNCYFPLQSY GFQPTNGVGYQPYR509 |_____| **(C480-C488)**
6	S-S 538-538	652.28<br>522.03	4<br>5	ND<br>ND	ND<br>ND	652.32<br>ND	652.29<br>522.04	652.24<br>522.00	652.29<br>522.02	538CVNF541-*HHHHHH*<br>| 538CVNF541-*HHHHHH*<br>**(C538-C538 homodimer, C-terminal)**
7	S-S 538-391	823.63<br>659.11	4<br>5	823.63<br>659.10	823.64<br>659.12	823.65<br>659.13	823.64<br>659.11	823.60<br>ND	823.65<br>659.10	538CVNF541-*HHHHHH*<br>| 387LNDLCFTNVYADSFVIR403<br>**(C538-C391)**

frontiers

Supplementary Table S4. O-glycosylation of six 500-liter batches of RBD (319-541)-His6 analyzed by ESI-MS. Nd, not determined.

Row	Code	*Theor m/z*	z	*Experimental m/z* (Monomer)			*Experimental m/z* (Dimer)			Description
				**5028/ P2109**	**5028/ P2110**	**5028/ P2111**	**5016/ P2116**	**5016/ P2117**	**5016/ P2118**	
1	R319 - R328	920.96<br>614.31	2<br>3	920.96<br>614.32	920.97<br>614.32	920.98<br>614.32	920.96<br>614.32	920.92<br>nd	920.94<br>614.31	319RVQP***T*** E***S***IVR328 +HexNAc:Hex:NeuAc (N-terminal, *O*-glycosylated at T323/S325)
2	-	934.95<br>623.64	2<br>3	934.95<br>623.64	934.96<br>623.65	934.95<br>623.65	nd<br>nd	nd<br>nd	934.97<br>623.64	319RVQP***T*** E***S***IVR328 +HexNAc:190Da:NeuAc (N-terminal, *O*-glycosylation T323/S325, Hex+28Da)
3	R319 - R328	1066.50<br>711.34	2<br>3	1066.53<br>711.35	1066.48<br>711.34	1066.50<br>711.36	1066.49<br>711.34	1066.46<br>nd	1066.52<br>711.35	319RVQP***T*** E***S***IVR328+HexNAc:Hex:NeuAc2 (N-terminal, *O-glycosylation* T323/S325)
4	-	1080.50<br>720.67	2<br>3	1080.48<br>720.68	1080.43<br>720.68	1080.53<br>720.69	nd<br>720.68	nd<br>nd	1080.48<br>720.68	319RVQP***T*** E***S***IVR328 +HexNAc:190Da:NeuAc2 (N-terminal, *O-glycosylation* T323/S325, Hex+28Da)
5	V320 - R328	616.33	2	nd	nd	nd	nd	616.29	nd	320VQP***T***E ***S***IVR328 +HexNAc (*O-glycosylation* T323/S325)
6	V320 - R328	697.36	2	697.37	697.36	697.37	697.33	697.31	697.37	320VQP***T***E ***S***IVR328 +HexNAc:Hex (*O-glycosylation* T323/S325)
7	V320- R328 + 638Da	-	2	833.89	833.94	833.93	nd	833.83	833.90	320VQP***T***E ***S***IVR328 + 638 Da
8	V320 - R328	842.90	2	842.92	842.91	842.92	842.91	842.85	842.90	320VQP***T***E ***S***IVR328 +HexNAc:Hex:NeuAc (*O-glycosylation* T323/S325)
9	-	856.90	2	856.90	856.90	856.94	856.91	856.85	856.90	320VQP***T***E ***S***IVR328 + HexNAc:190Da:NeuAc (*O-glycosylation* T323/S325, Hex+28Da)
10	V320 - R328	988.45	2	988.46	988.45	988.47	988.45	988.40	988.45	320VQP***T***E ***S***IVR328 +HexNAc:Hex:NeuAc2 (*O-glycosylation* T323/S325)
11	-	1002.45	2	1002.46	1002.45	1002.47	1002.44	1002.40	1002.46	320VQP***T***E ***S***IVR328 + HexNAc:190Da:NeuAc2 (*O-glycosylation* T323/S325, Hex+28Da)

12	-	-	2	1007.46	1007.39	1007.44	1007.42	1007.39	1007.45	320VQP***T***E ***S***IVR328 + 985 Da (*O-glycosylation* T323/S325)

Supplementary Table S5. The glycan structures of NP-HPLC chromatogram peaks were assigned using Glucose Unit (GU) measurements. The GU values for each peak were determined by calibrating with a glucose homopolymer ladder. The abundance of each specific glycan structure was quantified using the % peak area. RT, retention time.

Peak	RT	Area	%Area	Ladder	Structure		Theor. GU
1	78.688	155880	0.2802	5.45	G0	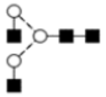	NA
2	83.437	266839	0.4797	5.89	G0F	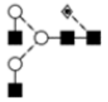	5.91
3	86.348	678149	1.2192	6.20	M5	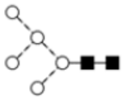	6.19
4	87.917	91047	0.1637	6.37	NA	NA	NA
5	89.518	39790	0.0715	6.55	NA	NA	NA

Supplementary Table S5. Cont**.**

Peak	RT	Area	%Area	Ladder	Structure		Theor. GU
6	90.696	141094	0.2537	6.68	G1F	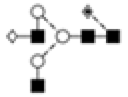	6.67
7	91.576	102172	0.1837	6.78		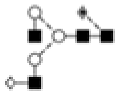	6.75
8	91.887	70867	0.1274	6.81	NA	NA	NA
9	93.920	357455	0.6426	7.04	NA	NA	NA
10	94.918	283417	0.5095	7.17	NA	NA	NA
11	96.062	267367	0.4807	7.32	NA	NA	NA
12	97.782	1620328	2.9130	7.54	G2F	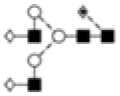	7.56
13	100.198	9854677	17.7165	7.85	G2FS1	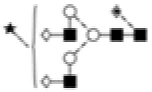	8.07
14	102.839	23380260	42.0325	8.22	G2FS2	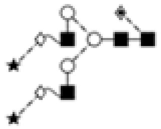	8.39
15	105.687	978900	1.7598	8.64		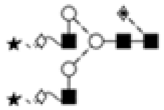	

Supplementary Table S5. Cont.

Peak	RT	Area	%Area	Ladder	Structure		Theor. GU
16	107.283	482148	0.8668	8.88	G3FS1	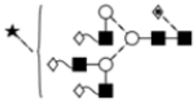	8.89
17	108.245	935237	1.6814	9.02	G3FS2	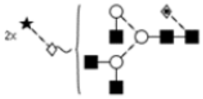	9.09
18	109.699	3764319	6.7674	9.27	G3FS3	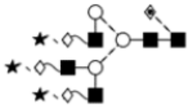	9.77
19	111.400	4684489	8.4217	9.55	G3FS3	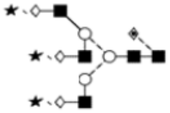	NA
20	113.898	39191	0.0705	9.97	NA	NA	NA
21	115.330	1923880	3.4587	10.24	G4FS1	NA	NA
22	116.511	2315532	4.1628	10.46	G4FS2	NA	NA
23	117.508	700191	1.2588	10.65	G4FS3	NA	NA
24	120.871	1920457	3.4526	11.32	G4FS4	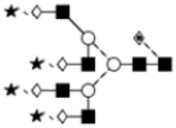	11.29
25	124.718	490576	0.8819	12.14	NA	NA	NA

NA: Not available

G0: GlcNAc2Man3GlcNAc2; G0F: GlcNAc2Man3GlcNAc2Fuc; G1F: GalGlcNAc2Man3GlcNAc2Fuc

M5: Man5; G2F: Gal2GlcNAc2Man3GlcNAc2Fuc; G2FS1: Neu5AcGal2GlcNAc2Man3GlcNAc2Fuc

G2FS2: Neu5Ac2Gal2GlcNAc2Man3GlcNAc2Fuc; G3FS1: Neu5AcGal3GlcNAc2Man3GlcNAc2Fuc

G3FS2: Neu5Ac2Gal3GlcNAc2Man3GlcNAc2Fuc; G3FS3: Neu5Ac3Gal3GlcNAc2Man3GlcNAc2Fuc

G4FS4: Neu5Ac4Gal4GlcNAc2Man3GlcNAc2Fuc

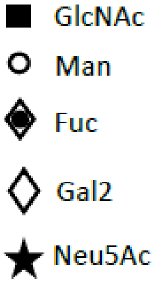

Supplementary Table S6. Stability at 2-8°C of three independent batches of purified RBD (319-541)-His6 monomer. Samples were collected at 0, 1, 2, 3, 6, 9, and 12 months to assess the stability of the protein over time. The data suggest that the purified RBD monomer remained stable over the course of 12 months when stored at 2-8°C. There was a slight decrease in the percentage of remaining protein over time, but this was within an acceptable range for a protein intended for vaccine use.

Parameter	Method	Batch	Time 0	Month 1	Month 2	Month 3	Month 6	Month 9	Month 12
Biological activity	RBD-specific ELISA	1	Complies	Complies	Complies	Complies	Complies	Complies	Complies
		2	Complies	Complies	Complies	Complies	Complies	Complies	Complies
		3	Complies	Complies	Complies	Complies	Complies	Complies	Complies
Purity	Percent of most abundant SDS-PAGE band (32 000 Mr)	1	100	96.53	100	100	100	100	100
		2	100	100	100	100	100	100	100
		3	100	100	100	100	100	100	100
	Percent of RBD dimer by SEC-HPLC	1	11.32	12.55	12.11	13.94	15.18	16.50	18.21
		2	16.25	17.71	17.28	19.66	20.35	21.83	22.73
		3	11.28	13.57	13.45	15.49	17.05	17.51	14.71
	Percent of RBD monomer by SEC-HPLC	1	88.68	87.43	87.85	86.06	84.32	83.31	81.4
		2	83.69	82.28	82.68	80.34	79.36	78.10	77.22
		3	88.72	86.39	86.39	84.51	82.50	82.41	85.24

pH	Potentiometry	1<br>2<br>3	7.3<br>7.2<br>7.3	7.3<br>7.2<br>7.3	7.3<br>7.2<br>7.2	7.3<br>7.2<br>7.3	7.2<br>7.2<br>7.2	7.2<br>7.2<br>7.1	7.2<br>7.2<br>7.2
Identity	Peptide mapping	1<br>2<br>3	Complies<br>Complies<br>Complies	ND<br>ND<br>ND	ND<br>ND<br>ND	Complies<br>Complies<br>Complies	Complies<br>Complies<br>Complies	ND<br>ND<br>ND	Complies<br>Complies<br>Complies
Protein concentration	Spectrophotometry	1<br>2<br>3	1.70<br>1.30<br>1.40	1.57<br>1.22<br>2.05	1.52<br>1.21<br>1.30	1.53<br>1.19<br>1.26	1.60<br>1.20<br>1.30	1.40<br>1.30<br>1.41	1.52<br>1.25<br>1.31
N-glycosilation	Percent of major N-glycosylation peaks separated by NP-HPLC		G2F: 3; 5; 3<br>G2FS1: 18; 26; 20<br>G2FS2: 36; 39; 35	ND	ND	G2F: 3; 3; 3<br>G2FS1: 19; 18; 18<br>G2FS2: 38; 40; 41	G2F: 1; 1; 2<br>G2FS1: 15; 15; 16<br>G2FS2: 43; 43; 42	ND	G2F: 2; 2; 3<br>G2FS1: 17; 15; 15<br>G2FS2: 41; 45; 43

ND: Not determined.

G2F:Gal2GlcNAc2Man3GlcNAc2Fuc   G2F:

G2FS1:Neu5Ac Gal2GlcNAc2Man3GlcNAc2Fuc   G2FS1:

G2FS2:Neu5Ac2 Gal2GlcNAc2Man3GlcNAc2Fuc   G2FS2:

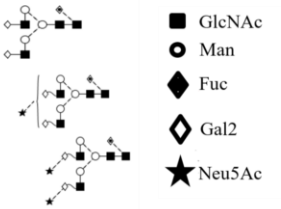

Supplementary Table S7. Stability at 2-8 ºC of three independent batches of purified RBD (319-541)-His6 dimer. Samples were collected at 0, 1, 2, 3, 6, 9 and 12 months to assess the protein stability over time.

Parameter	Method	Time 0	Month 1	Month 2	Month 3	Month 6	Month 9	Month 12
Biological activity	RBD-specific ELISA	Complies<br>Complies<br>Complies	Complies<br>Complies<br>Complies	Complies<br>Complies<br>Complies	Complies<br>Complies<br>Complies	Complies<br>Complies<br>Complies	Complies<br>Complies<br>Complies	Complies<br>Complies<br>Complies
Purity	Percent of most abundant SDS-PAGE band (50-75 000 Mr)	100<br>100<br>100	100<br>100<br>100	100<br>100<br>100	100<br>100<br>100	100<br>100<br>100	100<br>100<br>100	100<br>100<br>100
	Percent of RBD dimer by SEC-HPLC	97.63<br>97.05<br>97.57	96.70<br>96.37<br>99.86	94.08<br>94.15<br>95.24	95.11<br>95.03<br>95.24	90.46<br>91.58<br>90.95	92.44<br>91.93<br>92.13	91.31<br>90.01<br>91.45
	Percent of RBD monomer by SEC-HPLC	2.37<br>2.95<br>2.43	3.22<br>3.55<br>0	5.81<br>5.80<br>4.72	4.84<br>4.77<br>4.68	9.25<br>7.96<br>8.38	7.49<br>7.93<br>7.64	8.47<br>9.89<br>8.41
pH	Potentiometry	7.3<br>7.2	7.3<br>7.2	7.3<br>7.2	7.3<br>7.3	7.2<br>7.2	7.2<br>7.1	7.2<br>7.2

		7.3	7.3	7.2	7.3	7.2	7.2	7.2
Identity	Peptide mapping	Complies	ND	ND	Complies	Complies	ND	Complies
Protein concentration	Spectrophotometry	0.72<br>0.73<br>0.70	0.68<br>0.68<br>0.72	0.72<br>0.66<br>0.61	0.69<br>0.69<br>0.70	0.70<br>0.60<br>0.60	0.72<br>0.70<br>0.70	0.71<br>0.70<br>0.71
N-glycosylation	Percent of major N-glycosylation peaks separated by NP-HPLC	G2F: 4; 4; 4<br>G2FS1: 26; 24; 25<br>G2FS2: 44; 41; 41	NP	NP	G2F: 3; 3; 2<br>G2FS1: 18; 18; 17<br>G2FS2: 43; 42; 42	G2F: 2; 2; 3<br>G2FS1: 16; 17; 18<br>G2FS2: 43; 40; 37	ND	G2F: 3; 2; 3<br>G2FS1: 16; 18; 18<br>G2FS2: 43; 41; 41

ND: Not determined.

G2F:Gal2GlcNAc2Man3GlcNAc2Fuc G2F:

G2FS1:Neu5Ac Gal2GlcNAc2Man3GlcNAc2Fuc G2FS1:

G2FS2:Neu5Ac2 Gal2GlcNAc2Man3GlcNAc2Fuc G2FS2:

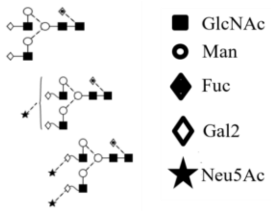

Supplement: Supplementary file 1 [file DataSheet1.pdf]
